# Phase I Trial of Consolidative Radiotherapy with Concurrent Bevacizumab, Erlotinib and Capecitabine for Unresectable Pancreatic Cancer

**DOI:** 10.1371/journal.pone.0156910

**Published:** 2016-06-23

**Authors:** Awalpreet S. Chadha, Heath D. Skinner, Jillian R. Gunther, Mark F. Munsell, Prajnan Das, Bruce D. Minsky, Marc E. Delclos, Deyali Chatterjee, Huamin Wang, Marilyn Clemons, Geena George, Pankaj K. Singh, Matthew H. Katz, Jason B. Fleming, Milind M. Javle, Robert A. Wolff, Gauri R. Varadhachary, Christopher H. Crane, Sunil Krishnan

**Affiliations:** 1 Department of Radiation Oncology, The University of Texas MD Anderson Cancer Center, Houston, Texas, United States of America; 2 Department of Biostatistics, The University of Texas MD Anderson Cancer Center, Houston, Texas, United States of America; 3 Department of Pathology, The University of Texas MD Anderson Cancer Center, Houston, Texas, United States of America; 4 Department of Surgical Oncology, The University of Texas MD Anderson Cancer Center, Houston, Texas, United States of America; 5 Department of Gastrointestinal Medical Oncology, The University of Texas MD Anderson Cancer Center, Houston, Texas, United States of America; The Ohio State University, UNITED STATES

## Abstract

**Purpose:**

To determine the safety, tolerability and maximum tolerated dose (MTD) of addition of erlotinib to bevacizumab and capecitabine-based definitive chemoradiation (CRT) in unresectable pancreatic cancer.

**Methods:**

Seventeen patients with CT-staged, biopsy-proven unresectable pancreatic cancer were enrolled between 3/2008 and 10/2010. Prior chemotherapy was permitted. Two patients each were enrolled at dose levels (DLs) 1–4 and 9 patients at DL 5. All patients received 50.4 Gy (GTV only) in 28 fractions with concurrent capecitabine, bevacizumab and erlotinib. Dose of each drug was escalated in 5 DLs using the continual reassessment method. Bevacizumab was escalated from 5mg/Kg q2weeks (DLs 1–4) to 10mg/Kg q2weeks (DL 5); daily erlotinib from 100mg/day (DLs 1–2) to 150 mg/Kg (DLs 3–5); and capecitabine from 400mg/m^2^ twice daily on days of radiation (DL 1) to 650mg/m^2^ (DLs 2–3) to 825 mg/m^2^ (DLs 4–5). Reassessment for potential resection was performed 6–8 weeks later.

**Results:**

Sixteen patients received gemcitabine-based chemotherapy prior to CRT. With a median clinical follow-up of 10 months, no grade 3 toxicities were observed in DLs 1–4. Three (33%) patients at DL 5 developed a grade 3 acute toxicity (2 diarrhea, 1 rash). No grade 4 or 5 toxicities were seen. DL 4 was selected as the MTD; therefore, the recommended doses in combination with radiation are: bevacizumab, 5mg/Kg q2weeks; erlotinib, 150 mg/Kg daily; and capecitabine, 825mg/m^2^ BID. Median survival was 17.4 months. Of the five patients who underwent resection, 4 were originally deemed locally advanced and 1 was borderline resectable. Three patients had excellent pathological response (2 complete response and 20% viable tumor) at surgery, and the 2 patients with complete response are still alive at 61 and 67 months of follow up with no local or distant failures.

**Conclusions:**

This chemoradiation regimen at the recommended dose levels is safe and tolerable for patients with unresectable pancreatic cancer and merits further evaluation.

## Introduction

Pancreatic cancer is the third most common gastrointestinal (GI) malignancy and has a 5 year overall survival (OS) rate of less than 5%.[1, 2] Unfortunately, the absence of validated screening biomarkers and/or imaging tools coupled with the inherent aggressiveness of the tumor prevent detection at an early stage, and at least half of all patients have radiographically detectable metastatic disease at diagnosis.[3] Margin-negative surgical resection is the only potentially curative treatment for prima facie non-metastatic patients, and even they have a 5 year OS rate of only 15–25%,[4] with high rates of both local and distant progression. Patients with unresectable disease are generally treated with chemotherapy followed by chemoradiation therapy; treatment outcomes are universally poor with a 5 year OS rate of less than 10%. In contrast to the commonly held belief that pancreatic cancers rapidly disseminate via hematogenous metastases to the liver or intra-abdominal spread along the peritoneal lining, a recent autopsy series suggested that close to one-third of patients with pancreatic cancer die due to locally destructive disease rather than distant metastasis. Thus, improving local control is critical to improve overall treatment outcomes for patients with unresectable pancreatic cancer. [5] The role of radiation therapy in optimization of local control is even more important in the current setting, where advances in combination cytotoxic chemotherapy have improved distant control.

In recent years, there have been advances in our understanding of the genetic, biochemical and cellular makeup of pancreatic cancer. Pancreatic cancers overexpress epidermal growth factor (EGFR), and the presence of this receptor has been shown to correlate with negative outcomes.[6–10] After ligand activation, members of the EGFR family dimerize, trans-autophosphorylate each other, and subsequently activate a wide variety of downstream signals controlling cell proliferation, resistance to apoptosis, invasion, angiogenesis and metastasis.[11] Erlotinib is an orally administered human EGFR tyrosine kinase inhibitor which prevents autophosphorylation of the receptor dimer and activation of downstream targets. The utility of this agent has been proven in the setting of locally advanced, unresectable or metastatic pancreatic cancer with a large randomized phase III trial of 569 patients that showed a modest overall survival benefit (6.24 vs. 5.91 months, p = 0.04) with the addition of erlotinib to gemcitabine.[12]

Increased levels of vascular endothelial growth factor (VEGF) expression in tumors has been correlated with increased rates of recurrence and metastasis.[13–15] Bevacizumab, a humanized anti-VEGF monoclonal antibody has been studied extensively for possible use as an anti-angiogenic agent in pancreatic cancers patients. Our group has previously demonstrated that addition of bevacizumab to concurrent capecitabine-based radiation is safe in a phase I trial of locally advanced pancreatic cancer (LAPC) patients.[16] Inhibition of VEGF signaling in combination with radiation is believed to enhance both the endothelial cell and tumor cell toxicity.[17] However, there is evidence to suggest that, in the context of VEGF blockade, angiogenic signaling can be mediated by other tyrosine kinase signaling cascades, notably EGFR.[18] We hypothesized that simultaneous targeting of VEGF and EGFR pathways, in combination with chemoradiation, would lead to increased response rates in patients with pancreatic cancer.

Therefore, we designed a phase I trial to assess the safety and tolerability of adding erlotinib and bevacizumab to capecitabine-based definitive chemoradiation for patients with unresectable pancreatic cancer.

## Methods

### Patient eligibility

This protocol was approved and monitored by the University of Texas MD Anderson Cancer Center institutional review board. A total of 17 patients with unresectable pancreatic cancer (borderline and locally advanced) treated at our institution were enrolled in this study from March 10, 2008 to October 25, 2010. Inclusion criteria are shown in Table 1 and exclusion criteria are shown in Table 2. A tumor was deemed locally advanced if it extended to the celiac axis, the superior mesenteric artery (SMA) (>180 degrees encasement) or the aorta or occluded the superior mesenteric -portal venous (SMV-PV) confluence, based on review of imaging. For borderline pancreatic cancer (BRPC), we used the MD Anderson Cancer Center (MDACC) definition of BRPC which included tumors with segmental occlusion of the SMV/PV confluence, < 180° abutment of the SMA and celiac axis and abutment or short segment encasement of the common hepatic artery, typically at the origin of the gastroduodenal artery.

**Table 1:** Inclusion criteria for enrollment in the study.

ECOG performance status of 0 or 1
> 18 years of age (no upper age limit)
Cytologic or histologic proof of adenocarcinoma of the pancreas
No radiographic or clinical evidence of metastatic disease at the time of study entry
Locally advanced, unresectable disease (Tumor extension to the superior mesenteric artery (SMA), celiac axis, occlusion of the superior mesenteric vein (SMV) or portal venous confluence)
Found to be unresectable at the time of laparotomy
Unresectability due to underlying medical problems
Patients may have received prior chemotherapy but not prior radiation therapy to the upper abdomen
Absolute neutrophil count (ANC) >1,500 cells/ul
Platelet count >100,000 cells/ul
Total bilirubin < 5mg/dL with adequate biliary decompression
Alanine aminotransferase (ALT) < 5 times the upper limit of normal.
BUN < 30 mg/dl
Creatinine < 1.5 mg/dl and creatinine clearance > 30ml/min

**Table 2:** Exclusion criteria.

Prior abdominal radiotherapy.
Imaging (CT or MRI) or endoscopic evidence of direct duodenal invasion by tumor.
Prior therapy with bevacizumab, cetuximab, or gefitinib.
Prior therapy with erlotinib is permitted unless the patient was taken off erlotinib due to treatment failure.
Prior severe infusion reaction (bronchospasm, stridor, urticaria and/or hypotension) to a monoclonal antibody.
Prior unanticipated severe reaction to fluoropyrimidine therapy or known hypersensitivity to 5–fluorouracil.
Proteinuria at baseline or clinically significant impairment of renal function as demonstrated by urine dipstick for proteinuria > 2+
Prior history of cancer within the last five years except for basal cell carcinoma of the skin or carcinoma in situ of the cervix.
Pregnant or lactating women.
Serious, uncontrolled, concurrent infection(s) requiring IV antibiotics
Clinically significant cardiovascular disease, arrthymia or peripheral vascular disease within 6 months of study entry
Psychiatric disorders rendering patients incapable of complying with the requirements of the protocol.
History or evidence upon physical examination of CNS disease
Prior history of pulmonary embolism or deep venous thrombosis.
Major surgical procedure, open biopsy, or significant traumatic injury within 28 days prior to study entry
Fine needle aspirations or core biopsies within 7 days prior to study entry
Lack of physical integrity of the upper gastrointestinal tract, malabsorption syndrome or inability to swallow
Known, existing uncontrolled coagulopathy (INR > 1.5)
Recent use of anti-coagulation other than low dose (1mg) coumadin, IV and LMW heparin
Current serious, nonhealing wound, ulcer, or bone fracture.
History of abdominal fistula, gastrointestinal perforation, or intra-abdominal abscess within 6 months prior to study entry
The presence of an organ allograft.

### Study design

The primary endpoint of the study was the safety and tolerability of erlotinib and bevacizumab in combination with capecitabine-based definitive chemoradiation in the study patient population. Secondary endpoints included radiographic and pathologic response rates, rate of margin-negative resection in patients deemed unresectable at initiation of treatment, disease-free survival, and OS.

A quality of life questionnaire was administered at baseline and weekly throughout treatment.

### Systemic therapy treatment plan

Patients were sequentially treated with 5 escalating dose levels (table 3), in cohorts of size 2 starting at the lowest dose. The maximum tolerated dose (MTD) was determined from these 5 dose combinations using the continual reassessment method.[19] Capecitabine was given orally twice a day on the days of radiation, with the dose increased from 400 mg/m^2^ in dose level 1, to 825 mg/m^2^ in dose level 5. Erlotinib was given orally once a day throughout the course of chemoradiation. The dose was increased from 100 mg in dose levels 1–2 to 150 mg in dose levels 3–5. Bevacizumab was administered intravenously every 2 weeks at 5mg/kg (dose levels 1–4) or 10mg/kg (dose level 5) for a total of 3 doses, starting on the first day of radiation.

**Table 3:** Study Schema.

Dose Level	Bevacizumab (mg/Kg q2w)	Capecitabine (mg/m2 bid)	Erlotinib (mg qd)
**1**	5	400	100
**2**	5	650	100
**3**	5	650	150
**4**	5	825	150
**5**	10	825	150

### Radiation therapy technique

Patients were treated over a period of 5–6 weeks to a total dose of 50.4 Gy in 28 fractions. The primary tumor and any clinically enlarged lymph nodes were targeted using either a 3-D conformal radiation therapy or intensity modulated radiation therapy (IMRT). For 3-D conformal planning, a 2–4 field technique was used (margin from tumor to block edge 2 cm radial, 3 cm cranial/caudal), and the total dose was prescribed to the 95% isodose line. Only one patient was treated with an IMRT boost; for this, a multiple beam technique was used to ensure 95% dose coverage to the GTV.

### Dose modifications for toxicity

Toxicities were evaluated using the National Cancer Institute Common Terminology Criteria for Adverse Events, version 3.0. Capecitabine was withheld in patients who developed grade 2 or greater neutropenia, hand-foot syndrome, mucositis or gastrointestinal (GI) toxicities unresponsive to medical management and was restarted following recovery to grade 1. The dose was adjusted based on the number of episodes of grade 2 or higher events: dose was reduced to 75% and 50% of starting dose after the first or second occurrence, respectively, and discontinued after the third occurrence. Once doses were reduced they were never increased at a later time. Bevacizumab infusion was interrupted for any grade 3 or greater toxicity suspected to be related to the drug; treatment was withheld until the toxicity resolved to grade 1 and then continued without dose adjustment. Erlotinib was withheld if the patients developed grade 3 diarrhea or GI bleeding, an intolerable rash or a pulmonary event possibly related to the drug (pending further evaluation) and was restarted at a reduced dose following recovering to grade 2 or lower toxicity. Dose re-escalation was allowed for intolerable rash but not for grade 3 diarrhea. Erlotinib was permanently discontinued if a patient developed grade 4 diarrhea, rash or if the pulmonary event was determined to be related to the drug.

### Patient follow-up

Restaging using pancreatic protocol CT, chest X-ray and CA19-9 along with surgical evaluation was performed 5–6 weeks after completion of chemoradiation. All patients with stable or responding disease were offered the option of either surgical resection (if technically resectable) with maintenance therapy or maintenance bevacizumab and erlotinib (at the same dose-level as used during chemoradiation) until disease progression. Further follow-up was scheduled every 2 months. Locoregional recurrence was defined as any recurrence at or adjacent to the initial primary site or in the regional lymph nodes as determined by abdominal-pelvic CT scans.

### Smad4 immunostaining of diagnostic specimens

Out of the 17 specimens examined, only 11 contained adequate tumor cellularity for analysis, six of these were pre-treatment fine needle aspirates. Immunohistochemical staining for SMAD4 was performed either on 5-μm unstained sections from biopsy FFPE blocks or cytology smears from fine needle aspiration specimens. Following deparaffinization, antigen retrieval was performed on the tissue sections at 100°C in a steamer containing 10 mmol Tris-EDTA buffer for 35 min. The sections were then washed and immersed in anti-SMAD4 antibody (Clone B-8, 1:100 dilution, Santa Cruz Biotechnology) at 35°C for 15 min. Subsequently, the sections were immersed in 3.0% hydrogen peroxidase at 35°C for 5 min to block the endogenous peroxidase activity. A polymer enhancer solution was then applied to the slides and incubated at 35°C for 8 minutes. The sections were then incubated with secondary Poly-HRP anti-mouse/anti-rabbit immunoglobulin at 35°C for 8 min. Diaminobenzidine (DAB) was used as a chromogen and DAB enhancer was applied, and hematoxylin was used for counterstaining. Each specimen slide was scored as either Smad4 (Dpc4) positive or Smad4 (Dpc4) negative by a cytopathologist.

### Statistical design

Standard response criteria were used to assess tumor response. OS and disease-free survival were estimated using the Kaplan-Meier method. OS was calculated from the start of chemoradiation to the time of death or last follow-up on record if death was not observed. Disease-free survival was calculated from the start of chemoradiation to the date of documented disease progression (locoregional or distant). If no progression was observed, patients were censored at the date of last follow-up. Advancement of dose levels was determined based on the continual reassessment method where the maximum acceptable DLT rate was 25%.[20] The *a priori* DLT probabilities for the 5 dose levels were assumed to be 0.03, 0.05, 0.10, 0.15, and 0.20. An added measure of safety was employed such that if there was more than a 0.95 probability that the DLT rate of the first dose exceeded 25% the study would be stopped.

## Results

### Patient characteristics

**Fig 1:**
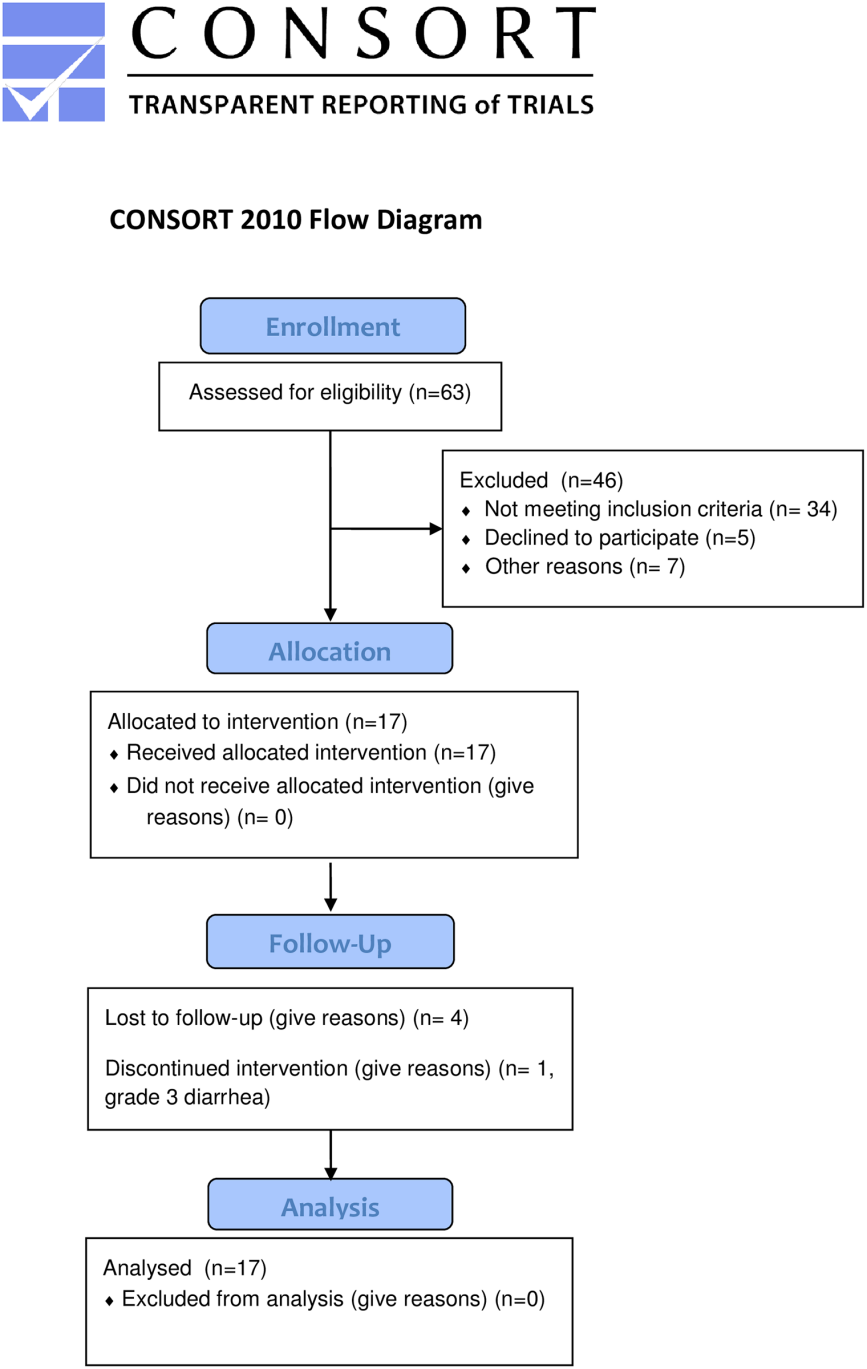
CONSORT flow diagram of phase I trial.

Patient and tumor characteristics are shown in Table 4 and Fig 1 details patient progression through the trial. Most patients had locally advanced tumors at the time of protocol entry (76.4%), with two patients (11.8%) having borderline resectable tumors due to venous involvement, one patient (5.9%) unable to undergo surgery secondary to comorbidity, and one patient (5.9%) with gross residual disease following an R1 resection. The majority (94.1%) of patients were treated with gemcitabine-based chemotherapy prior to chemoradiation, with a median of 4 cycles (range 3–10). After chemoradiation, unresectable patients were treated with maintenance therapies including erlotinib and bevacizumab (2 patients); gemcitabine and cisplatin (2 patients); FOLFOX (2 patients; of these, one was treated with palliative intent due to hepatic metastasis); capecitabine and erlotinib (1 patient); capecitabine and oxaliplatin (1 patient); single agent erlotinib (1 patient); palliative surgery (1 patient); and single agent 5-FU (1 patient). For the patients who underwent surgery (5 patients), maintenance therapies included single agent capecitabine (1 patient), gemcitabine and cisplatin (2 patients), and observation (2 patients). The median clinical and radiological follow up times from completion of chemoradiation were 10 months (range, 1–69 months) and 9 months (range, 1–69 months) respectively.

**Table 4:** Patient characteristics.

Characteristic	No.	%
**Age (years)**		
Median	64	
Range	44–77	
**Follow up from XRT end date (months)**		
Median	10	
Range	1–69	
**Tumor diameter prior to XRT (cm)**	3.2	
Median	3.2	
Range	1.8–5	
**CA-19-9 prior to XRT (U/ml)**		
Median	127.5	
Range	1–2933	
**Gender**		
Male	12	71
Female	5	29
**Race**		
Caucasian	15	88
African-American	1	6
Hispanic	1	6
**KPS**		
100	2	12
90	15	88
**Tumor stage**		
T2	1	5.9
T3	6	35.3
T4	10	58.8
**Nodal stage**		
N0	5	29.4
N1	12	70.6
**Surgical status**		
Borderline resectable	2	11.8
Locally advanced	13	76.4
Co-morbidity	1	5.9
Localized disease post-R1 resection	1	5.9

### Toxicity

Toxicities separated by dose level (DL) are shown in Table 5. No grade 3 toxicities were observed in dose levels 1–4, while three patients in group 5 had grade 3 toxicity. One patient developed an acneiform rash that covered >30% of his body after 10 fractions of radiation. The patient was prescribed oral antibiotics and erlotinib was stopped for five days. The rash improved and the patient resumed erlotinib at fraction 15. Two patients developed grade 3 diarrhea. Neither patient required hospitalization. One patient required discontinuation of capecitabine two days prior to therapy completion and a dose reduction in erlotinib; this patient also experienced grade 2 HFS. The second patient had capecitabine discontinued after fraction 23 and erlotinib discontinued after fraction 25. For both patients, the diarrhea resolved by the end of radiation. For patients with GI toxicity (three patients with grade 2 diarrhea and four patients with grade 2 nausea), the dose of capecitabine was reduced only in one patient with grade 2 nausea; in others dose reduction was not required as the nausea and diarrhea was responsive to medical management (as specified per methods). Based on these results, DL 4, with a posterior probability of DLT of 0.122, was selected as the maximum tolerated dose. DL5 had a posterior probability of 0.263 which was slightly greater than the pre-defined maximum acceptable limit of 0.25. Toxicities separated by type are shown in Table 6. The most common toxicities overall included: grade 1 anorexia (94.1%), nausea (88.2%), fatigue (76.5%), diarrhea and rash (70.6% each). Quality of life metrics were compared for patients in DL1-4 and DL5 and were non-significant.

**Table 5:** Toxicity by dose level.

	Total number of patients		Maximal toxicity grade					
			I		II		III	
Dose level	N	%	N	%	N	%	N	%
1	2	11.8%	0	0.0%	2	11.8%	0	0.0%
2	2	11.8%	0	0.0%	2	11.8%	0	0.0%
3	2	11.8%	1	5.9%	1	5.9%	0	0.0%
4	2	11.8%	2	11.8%	0	0.0%	0	0.0%
5	9	52.9%	2	11.8%	4	23.5%	3	17.6%

**Table 6:** Individual toxicity grade.

	Toxicity grade					
Toxicity	I		II		III	
	N	%	N	%	N	%
Acne	1	5.9%	-	-	-	-
Allergy	1	5.9%	-	-	-	-
Alopecia	1	5.9%	-	-	-	-
Anorexia	16	94.1%	3	17.6%	-	-
Diarrhea	12	70.6%	3	17.6%	2	11.8%
Fatigue	13	76.5%	4	23.5%	-	-
H/F syndrome	3	17.6%	1	5.9%	-	-
Nausea	15	88.2%	4	23.5%	-	-
Neuropathy	2	11.8%	-	-	-	-
Pain	-	-	1	5.9%	-	-
Dermatitis	1	5.9%	1	5.9%	-	-
Rash	12	70.6%	4	23.5%	1	5.9%
Scalp tenderness	1	5.9%	-	-	-	-
Stomatitis	3	17.6%	1	5.9%		
Urinary frequency	1	5.9%	-	-	-	-
Vomiting	5	29.4%	-	-	-	-

### Radiographic response

All seventeen patients had stable disease based on RECIST criteria, and no correlation with dose levels was observed.

### Surgical resection and pathologic response

Five patients (29.4%) were felt to be surgically resectable following radiotherapy based on radiographic evidence of sufficient disease response. At time of diagnosis, only one was deemed borderline resectable; the remaining patients were locally advanced. Four of these patients received dose level 5, and one patient received dose level 2. Of the patients who were able to undergo resection, two patients had a complete pathologic response, one patient had <20% viable tumor, one patient had 90% viable tumor and one patient had a resection at an outside facility (unknown response to therapy). All patients had negative resection margins. Of the 2 patients with a complete pathological response, there have been no disease recurrences or deaths at follow-up of 61 and 67 months after completion of chemoradiation. The remaining three resected patients developed distant metastases at 5, 12 and 38 months.

### Survival outcomes and patterns of progression

**Fig 2:**
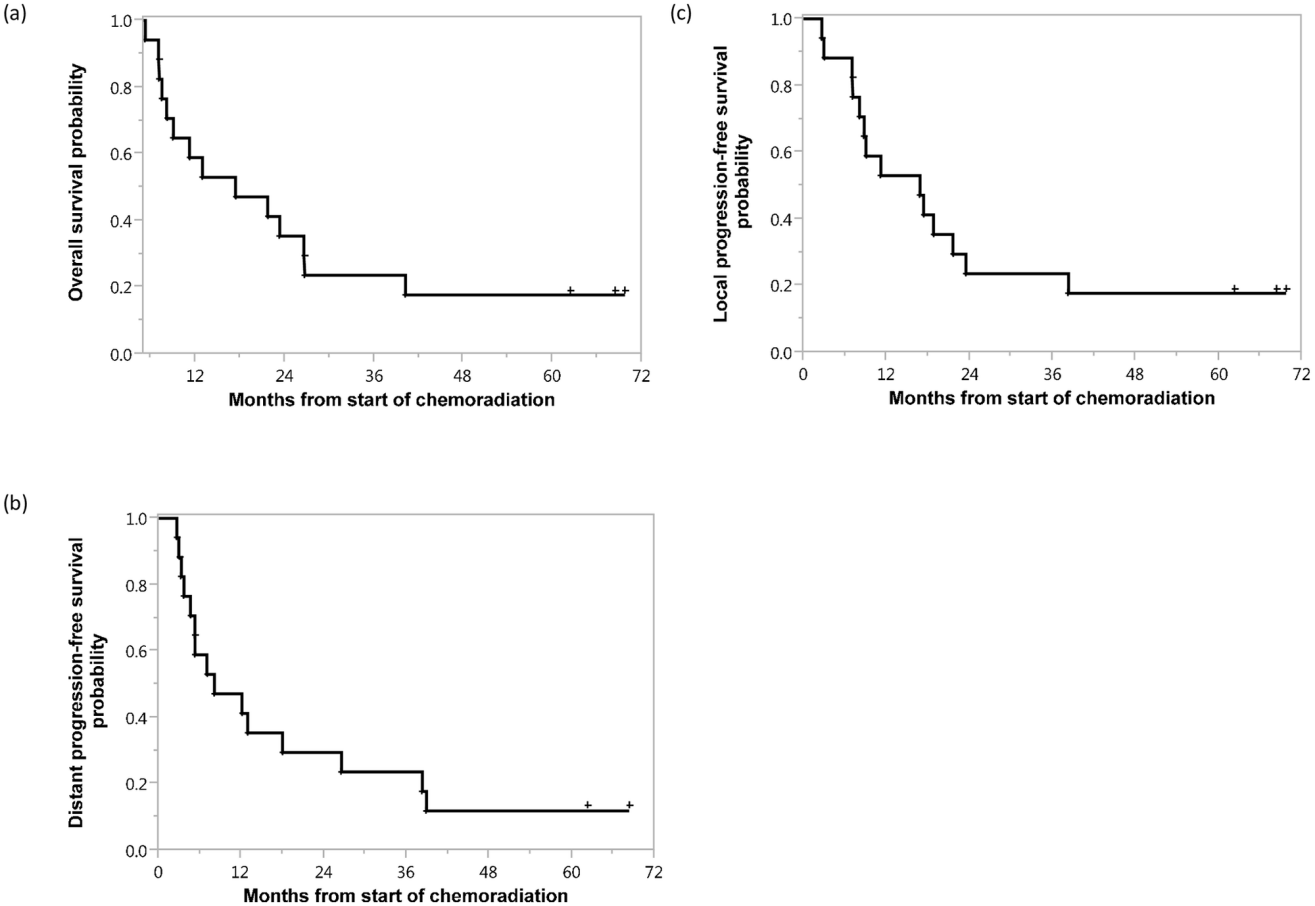
Kaplan-Meier estimates of (a) overall survival, (b) distant progression-free survival and (c) local progression-free survival from start of chemoradiation therapy.

Median survival from start of chemoradiation was 17.4 months (95% CI, 7.5 to 26.7 months) with a 1 year overall survival of 59% (Fig 2a). Thirteen (76%) patients had progressive disease. The median progression-free survival was 8.1 months (95% confidence interval, CI, 3.3 to 23.5 months). The first site of progression was locoregional in three (18%) patients, distant in seven (41%) patients, and synchronous locoregional and distant sites in three (18%) patients. The other four patients (23%) did not have any evidence of locoregional or distant disease progression at the time of last follow-up. Median time to distant failure was 12.1 months (95% CI, 3.7 to 38.9 months), with a 1 year freedom from metastasis rate of 55%. The median distant progression-free survival was 8.1 months (95% CI, 3.7 to 26.6 months) (Fig 2b). The most common initial site for distant metastasis was the liver (7 patients, 41%). Other initial sites of distant metastasis included: lung and peritoneal carcinomatosis (2 patients each, 12%) and non-regional lymphadenopathy (1 patient, 6%). Median time to locoregional progression was 21.6 months (95% CI, 8.8 to 16.9 months) with a 1 year freedom from progression rate of 77%. The median local progression-free survival was 16.9 months (95% CI, 7.2 to 23.5 months) (Fig 2c). All sites of locoregional progression were within the treatment field.

### SMAD-4 expression and survival outcomes

The pretreatment Smad4 (Dpc4) status was available for six patients, of which, two were scored as Smad4 (Dpc4) intact (immunohistochemically positive). Of these two patients, one had a complete pathological response following surgical resection and the other had synchronous loco-regional and distant progression. Among the 4 patients with loss of Smad4, one progressed distantly as their first site of failure, two progressed loco-regionally, and one had no evidence of loco-regional or distant failure at last follow-up. In our study, Smad4 expression was not associated with a particular pattern of disease progression. Intact Smad4 expression was, surprisingly, associated with a trend towards poor overall survival (9 vs. 21.7 months, p = 0.82) and progression free survival (3.3 vs. 8.8 months, p = 0.93). However, the sample size is too small to draw any definitive conclusions. Smad4 status of patients enrolled on the current RTOG 1201 protocol will provide additional information on the prognostic value of this marker. Furthermore, where possible, the use of core biopsies instead of fine needle aspirates may overcome some of the challenges with staining sparse tissue in cytology blocks remaining after processing for clinical assessment.

## Discussion

The advances in our understanding of the molecular mechanisms responsible for transformation and progression of pancreatic cancer have led to renewed interest in the development of novel, rationally-designed, molecularly-targeted therapies. These have been evaluated in several clinical trials in combination with standard chemoradiation regimens.[12, 16, 21–24] This phase I study demonstrates the safety and tolerability of combining erlotinib and bevacizumab with capecitabine-based chemoradiotherapy for patients with unresectable pancreatic cancer. Treatment was tolerated well with no unexpected increases in the frequency or severity of toxicities. The maximum tolerated dose was determined as erlotinib 150mg/day and bevacizumab 5mg/Kg every two weeks when given with capecitabine-based chemoradiation therapy. Therefore, the recommended doses in combination with radiation are bevacizumab: 5mg/Kg q2weeks, erlotinib: 150 mg/Kg daily, and capecitabine 650mg/m^2^ BID. The median survival was 17.4 months from the start of chemoradiation therapy (19.4 months from the date of diagnosis) which is comparable to some of the better survival outcomes reported in literature. Remarkably, four of the five patients who were able to undergo surgery following chemoradiation were locally advanced at time of diagnosis; of these, two had a complete pathological response and one patient had <20% viable tumor cells. Despite these promising outcomes, the significance of improving CRT outcomes by adding molecular targeted agents remains unclear when viewed within the context of recent evidence from the LAP07 trial which showed no benefit from the addition of CRT following induction chemotherapy (chemotherapy alone vs. chemotherapy followed by CRT, 16.5 vs. 15.3 months, p = 0.83) or of the addition erlotinib to gemcitabine (gemcitabine vs. gemcitabine plus erlotinib, 13.6 vs. 11.9 months, p = 0.09). Consequently, any benefit from addition of molecular agents would be limited to subsets of patients, if they are readily defined, who benefit from consolidative CRT in the first place.

Our group previously investigated the combination of bevacizumab with concurrent capecitabine and radiation therapy in a phase I trial and reported no additional grade 3 or higher gastrointestinal toxicity.[16] This approach was further tested in a multi-institutional phase II trial, RTOG 0411. Although bevacizumab-related adverse events were uncommon, there was no improvement in the 1-year overall survival rate.[21] The addition of erlotinib to conventional chemoradiation regimen for resectable pancreatic cancer was subsequently tested in a phase II study by Herman et al. This study showed that the combination of erlotinib (100mg/day) with capecitabine and radiation was relatively well-tolerated with overall grade 3 and 4 toxicity rates of 31% and 2% respectively.[22] Similarly, Duffy et al. in a phase I trial showed that the combination of erlotinib with gemcitabine and radiation in LAPC was relatively well-tolerated with grade 3 rash seen in 2 (14%) patients and grade 3 GI toxicity in 3 (21%) patients.[23] The MTD of erlotinib reported in this study was 100mg/day. Patients had a median survival of 18.7 months. There are phase I and II clinical data supporting the combination of bevacizumab and erlotinib alone or in combination with cytotoxic chemotherapy in a variety of other disease sites like rectal cancer and non-small cell lung cancer.[25–27] The present trial, to our knowledge, is the first to simultaneously evaluate the safety of adding two targeted agents, erlotinib and bevacizumab, to capecitabine-based chemoradiation in unresectable pancreatic cancer.

Pancreatic cancer cells overexpress a variety of growth factors and cytokines which confer a survival advantage by supporting self-sufficiency in nutritional and proliferative signals, evasion of apoptosis, and promotion of angiogenesis, invasion and dissemination. It has been proposed that blockade of a single pathway may not be capable of sufficiently deterring tumor growth, as acquired resistance mechanisms utilize redundant pathways. Thus, simultaneous treatment with bevacizumab and erlotinib, two agents working through different pathways involved in tumor growth, should, in theory, improve outcomes. Preclinical studies in xenograft models have supported this hypothesis, demonstrating that the combination of bevacizumab and erlotinib results in greater efficacy than either agent alone.[18, 28–30] Moreover, the antiangiogenic action of bevacizumab has been shown to stabilize tumor vasculature and enhance radiation response through improved tumor oxygenation.[31, 32] Furthermore, because there is little to no overlap in toxicity profile between the two agents, the combination appears to be well-tolerated and may provide a substantial benefit to patients who are unable to receive traditional cytotoxic therapy.

Our study was successful in determination of the MTD and recommended dose levels of erlotinib and bevacizumab when combined with capecitabine-based chemoradiation therapy. Only two patients (22%) treated at DL5 developed grade 3 diarrhea requiring dose adjustment or discontinuation of erlotinib and capecitabine. This is comparable to the results of RTOG 0411 (22%) study.[21] None of our patients developed grade 4 or 5 toxicity and overall only 18% had grade 3 toxicity at DL5. The 1 year overall survival rate of 59% is also comparable to the previously reported rates in RTOG 0411 (47%) and RTOG 9812 (43%) trials.[21, 33] These results provide encouraging evidence that bevacizumab and erlotinib may be safely combined with capecitabine and radiation. Five (29%) patients were successfully able to undergo surgical resection following chemoradiation. The two patients who had a complete pathological response had no recurrences at 5 years of follow-up, a finding similar to other studies showing improved survival in patients with complete response after chemoradiation.[34] Interestingly, the Smad4 expression in our study did not correlate with the pattern of disease progression. However, given the small number of patients evaluated and few patients with disease progression at time of evaluation, it is difficult to draw definitive conclusions from this cohort.

In summary, at the recommended dose levels, the addition of erlotinib and bevacizumab to capecitabine-based chemoradiotherapy is safe and tolerable in patients with unresectable pancreatic cancer. The rate of acute toxicity was minimal and similar to those observed in previous trials. In addition, the promising survival outcomes and high rate of conversion to resectability at the higher dose levels suggest that this strategy of dual inhibition of growth factor receptor pathways during chemoradiotherapy warrants continued evaluation in a larger study.

## Supporting Information

S1 File Trend Statement Checklist. (PDF)

S2 File Informed Consent Document. (PDF)

S3 File Protocol Page. (PDF)
