# Supplementary material for: Phase I Trial of Consolidative Radiotherapy with Concurrent Bevacizumab, Erlotinib and Capecitabine for Unresectable Pancreatic Cancer
Source: PLoS One. 2016 Jun 23;11(6):e0156910. doi: 10.1371/journal.pone.0156910 (PMC4919049; doi:10.1371/journal.pone.0156910)
Supplement: S2 File — (PDF) [file pone.0156910.s002.pdf]

THE UNIVERSITY OF TEXAS
MD Anderson ~~Cancer~~ Center

Informed Consent

Please Do Not Use for Patient Consent

Go to the PDOL Homepage to access the Informed Consent Printer Database

INFORMED CONSENT/AUTHORIZATION FOR PARTICIPATION IN RESEARCH WITH OPTIONAL PROCEDURES

Phase I Trial of preoperative radiotherapy with concurrent bevacizumab, erlotinib and capecitabine for locally advanced pancreatic cancer 2007-0044

Study Chair: Sunil Krishnan

1. ____________________________ ________________________
Participant's Name Medical Record Number

You are being asked to take part in this clinical research study at The University of Texas M. D. Anderson Cancer Center ("M. D. Anderson"). This consent form explains why this research study is being performed and what your role will be if you choose to participate. This form also describes the possible risks connected with being in this study. After reviewing this information with the person responsible for your enrollment, you should know enough to be able to make an informed decision on whether you want to participate in the study.

You are being asked to take part in this study because you have pancreatic cancer.

DESCRIPTION OF RESEARCH

2. PURPOSE OF STUDY

The goal of this clinical research study is to find the highest tolerable dose of capecitabine, erlotinib hydrochloride, and bevacizumab that can be given in combination with radiation to patients with pancreatic cancer.

Optional Procedures: You will be asked to have additional blood drawn. This blood will be used to learn if and how the combination of radiation, capecitabine, erlotinib, and bevacizumab may affect the tumor.

3. DESCRIPTION OF RESEARCH

The Study Drugs

Bevacizumab is designed to prevent or slow down the growth of cancer cells by blocking the growth of blood vessels.

Capecitabine and **erlotinib hydrochloride** are designed to interfere with the growth of cancer cells.

Screening Tests

Before you can receive the study drugs, you will have "screening tests" to help the doctor decide if you are eligible to take part in this study. The following tests will be performed:

- You will have a complete physical exam.
- Blood (about 2 tablespoons) will be drawn for routine tests.
- You will have chest x-rays and computed tomography (CT) scans of the abdomen (stomach area).
- You will complete a questionnaire about how you are feeling and any symptoms you may be experiencing. It should take about 10 minutes to complete.
- Women who are able to have children must have a negative urine pregnancy test.

Study Drug Dose Level

If you are found to be eligible to take part in the study, you will begin receiving capecitabine, erlotinib hydrochloride, and bevacizumab. The dose you receive will be based on how many participants have been enrolled before you, and on the safety data that are available. The first group of enrolled participants will be given low doses of capecitabine, erlotinib hydrochloride, and bevacizumab. If no intolerable side effects occur, the next group will be enrolled at a higher dose level. This process will continue until researchers find the highest dose of capecitabine, erlotinib hydrochloride, and bevacizumab that can be given without intolerable side effects occurring. The study doctor will tell you what dose you will be receiving and how it compares to the doses other participants have received.

Study Drug Administration

On Days 1, 14, and 28, you will receive bevacizumab through a needle in your vein. Your first infusion will last about 90 minutes. If you tolerate the drug well, the next infusion will last about 60 minutes. If the 60-minute infusion is well tolerated, all

other infusions will last about 30 minutes.

On each day that you receive radiation, you will take capecitabine and erlotinib hydrochloride by mouth in the morning and evening with food.

Radiation
You will receive radiation once a day on Monday through Friday, excluding holidays. This schedule will be continue for 5 1/2 weeks or 28 doses.

Study Visits
Every week while you are on study, you will have the following tests and procedures performed:

- You will have a physical exam.
- Blood (about 2 teaspoons) will be drawn for routine tests.
- You will be asked about any side effects you may be experiencing.
- You will repeat the same health questionnaire that you filled out at screening.

Bevacizumab and Surgery
If at any time during the study the tumor can be removed surgically, you will have surgery. A separate consent form would be used. Because bevacizumab may slow the healing of wounds, study participants may not have surgery within 10 weeks after the last bevacizumab infusion.

Length of Study
You will remain on study for up to 5 1/2 weeks. You will be taken off-study early if the disease gets worse or intolerable side effects occur.

End-Of-Study Visit
Four (4) to 6 weeks after you finish radiation, you will have an end-of-study visit with the following tests and procedures performed:

- You will have a complete physical exam.
- Blood (about 2 tablespoons) and urine will be collected for routine tests.
- You will have chest x-rays and CT scans of the abdomen.
- You will repeat the health questionnaire.

Additional Experimental Therapy
If you appear to be benefitting from the experimental therapy, the study doctor may decide to continue your experimental therapy after the end-of-study visit. This would be daily erlotinib hydrochloride, with bevacizumab infusions every 2 weeks unless the disease gets worse or intolerable side effects occur. You would have study visits once a month, with the same procedures as you did during the weekly study visits (except for the questionnaires).

This is an investigational study. Capecitabine, bevacizumab, and erlotinib hydrochloride are FDA approved and commercially available. The use of capecitabine and bevacizumab for pancreatic cancer and in combination with

erlotinib hydrochloride is investigational. At this time, the 3-drug combination is being used in research only.

Bevacizumab and erlotinib hydrochloride will be provided to you free of charge during the study. Capecitabine may be provided to you free of charge during the study if the cost of the drug is not covered by your insurance provider. All of the study tests will be performed free of charge. You will receive free parking for visits during the study-related radiation.

Up to 30 patients will take part in the study. All will be enrolled at M. D. Anderson.

Optional Procedures: If you agree, blood (about 1 tablespoon per draw) will be drawn before the start of radiation, during radiation (within 3 days of the first and third doses of bevacizumab), and at the end-of-study visit. This blood will be used to learn if the combination of radiation, capecitabine, erlotinib, and bevacizumab is having an effect on the tumor and what type of effect it may be having.

You do not have to agree to take part in the optional procedures in order to be enrolled in this study.

4. RISKS, SIDE EFFECTS, AND DISCOMFORTS TO PARTICIPANTS

While on this study, you are at risk for side effects. These side effects will vary from person to person. The more commonly occurring side effects are listed in this form, as are rare but serious side effects that the drugs are known to cause. You should discuss these with the study doctor. You may also want to ask about uncommon side effects that have been observed in small numbers of patients but are not listed in this form. Many side effects go away shortly after you stop receiving the study drugs, but in some cases side effects may be serious, long-lasting or permanent, and may even cause death.

Radiation therapy, capecitabine, **erlotinib hydrochloride**, and **bevacizumab** each may cause low blood cell counts (white blood cells, red blood cells, and platelets). This means that while you are on study, there is more of a chance of getting an infection, including pneumonia. You may become anemic and/or have problems with bleeding, bruising, fatigue, and/or shortness of breath. You may need a blood transfusion.

Radiation therapy may cause severe nausea, vomiting, diarrhea, and/or fatigue. Dehydration may occur. Delayed side effects may include intestinal damage.

Capecitabine may cause generalized swelling and/or fluid build-up in the lungs, torso, abdomen, legs, arms, and/or in the membrane covering the heart. It may cause episodes of irregular heartbeat, changes in heart rhythm, fast heartbeat, slow heartbeat, sudden stopping of the heartbeat, heart failure, and/or heart attack. It

may cause chest pain, nosebleeds, a blood clotting disorder, blood clots in a deep vein, and/or stroke. It may cause enlargement of the heart muscle, which can lead to heart failure. It may cause inflammation of the veins and/or blood vessels, which can lead to blood clots and/or bruising. It may cause inflammation of the muscular part of the heart. It may cause jaundice, giving the eyes and skin a yellow color.

Capecitabine may cause fatigue, fever, pain, headache, tiredness, mood changes, dizziness, impaired balance, and/or difficulty sleeping. It may cause depression, loss of consciousness, confusion, brain disease (encephalopathy), ECG changes, and/or sedation. It may cause you to feel irritable. It may cause difficulty in forming words and/or speaking them. It may cause the need to urinate frequently in the night. It may cause high levels of fat in the blood.

Capecitabine may cause hand-foot syndrome (a condition causing pain, swelling, numbness, tingling, and/or redness of the hands and feet). It may cause inflammation of the skin, nail disorder, skin rash, skin and/or nail discoloration, hair loss, and/or internal scarring. It may cause abnormal redness of the skin, excessive sweating, sores on the skin, skin reactions (irritation and redness) from sunlight exposure, itching, and/or a rash that is like a severe sunburn (called "radiation recall").

Capecitabine may cause dehydration, thirst, and/or hot flashes. It may cause low levels of potassium and/or magnesium in the blood, which can lead to weakness and/or abnormal heart rate. It may cause diarrhea, nausea, vomiting, constipation, vomiting of blood, and/or pain in the abdomen. It may cause decreased appetite, increased appetite, a digestive disorder, mouth discomfort, mouth sores, upset stomach, and/or changes in taste. It may cause an abnormal enlargement of the abdomen area inside the body. It may cause weight loss, weight gain, intestinal blockage, a stomach ulcer, hoarseness, difficulty swallowing, expansion of the colon, bloating, and/or pain in the rectum. It may cause inflammation of organs including the mouth, voice box, stomach lining, large intestine lining, stomach, intestines, and/or esophagus.

Capecitabine may cause high levels of a bile pigment in the blood, which can cause yellowing of the skin. It may cause blockage of the flow of bile from the liver, scarring of the liver, liver failure, and/or abnormal liver tests, which could mean liver damage. It may cause inflammation of the liver, which can cause liver failure.

Capecitabine may cause a general tickling and/or tingling sensation. It may cause muscle pain in the chest and/or back, pain in the arms and/or legs, general muscle pain, bone pain, abnormal muscle movements, and/or muscle twitching. It may cause weakness, joint stiffness, joint pain, and/or arthritis (joint inflammation). It may cause pain or weakness associated with damage to parts of the nervous system. It may cause eye irritation, abnormal vision, inflammation of the eyelid (also called "pink eye"), inflammation of the eye, and/or narrowing of the tear ducts that drain the eyes.

Capecitabine may cause decreased kidney function, difficulty breathing, cough, asthma, inflammation of the airways, pneumonia, wheezing, sore throat, blood clots in the lung, and/or noisy congested breathing. It may cause you to cough up blood. It may cause a viral and/or fungal infection, which may occur the mouth with mouth sores. It may cause a flu-like illness and/or a severe allergic reaction.

Capecitabine may also interact with Coumadin (warfarin), thinning the blood and increasing the risk of bleeding.

Bevacizumab may cause severe bleeding, including bleeding in the lungs, stomach, intestines, brain, or other parts of the body. It may cause a wound at a surgical connection and/or blood in the urine. Bevacizumab may cause vomiting (including vomiting of blood), decreased supply of blood to a body part (such as the heart, brain, or legs), delayed and/or poor wound healing, and/or coughing up blood. Bevacizumab may cause blood clots. The clots can occur in the veins or arteries that supply blood to the brain, heart, lungs, or other organs, which may cause stroke and/or heart attack. Heart failure may occur.

Bevacizumab may cause headaches, dizziness, confusion, weakness, pain associated with damage to parts of the nervous system, bleeding in the space surrounding the brain, and/or an abnormal manner of walking. It may cause brain disease (encephalopathy), including reversible posterior leukoencephalopathy syndrome, a disease of the white matter of the brain, which may lead to blindness. It may cause a hole to form in the nasal septum (the wall dividing the nasal cavity into halves).

Bevacizumab may cause chest pain, a decrease in heart function, and/or worsening of fluid collection surrounding the heart and/or within the tissues of the lung. It may cause rapid heartbeat or slow heartbeat. It may cause cough, a lack of oxygen in the tissues, an infection in the breathing passageways, and/or changes to the voice.

Bevacizumab may cause low blood pressure, high blood pressure, fainting, and/or seizure. In most patients, blood pressure can be controlled with routine medications taken by mouth while bevacizumab is continued. However, uncontrolled high blood pressure and high blood pressure resulting in disturbance of organ function may occur.

Bevacizumab may cause kidney damage. It may cause abnormally high values in liver blood tests, which may mean liver damage or liver failure. It may cause the abnormal presence of proteins in the urine, which may be due to kidney damage. It may cause watery eyes and/or nasal stuffiness. It may cause skin rash, shivering, chills, and/or fever. It may cause an allergic reaction, including a rapid onset of difficulty breathing and/or hives.

Bevacizumab may cause infected pockets of fluid in the abdominal area, irritation

and redness of the large intestine, and/or abnormal connections or passageways between organs or vessels that normally do not connect. It may cause intestinal blockage, abdominal pain, diarrhea, nausea, and/or loss of appetite. It may cause constipation, inflammation of the mouth, upset stomach, taste disorder, gas, and/or dry mouth. It may cause inflammation of the lining of the large intestine, death of tissue in the intestines, intestinal blockage, vein blockage in the abdomen, and/or inflammation of the membranes lining the abdomen and lungs. It may cause bowel perforation and/or bowel anastomotic dehiscence. Bowel perforation occurs when the wall of the stomach, small intestine or large intestine develops a hole through its entire thickness, which can cause leakage of the contents of these organs and lead to infection. Bowel anastomotic dehiscence is leakage from intestines that have been joined together, which can cause inflammation of the lining in the abdomen.

Bevacizumab may cause generalized pain, joint pain, and/or pain at the tumor site. It may cause a condition in which the lens of the eye becomes cloudy. Bevacizumab may also block VEGF. VEGF is important for the formation of new blood vessels at sites of low blood flow. Low blood flow exists in conditions such as coronary artery disease and cerebrovascular disease (abnormality of the brain resulting from blood vessel problems). Prolonged blockage of VEGF with bevacizumab could worsen any preexisting heart or cerebrovascular disease.

Bevacizumab may cause hair loss, dry skin, skin discoloration, nail disorder, and/or sores on the skin. It may cause skin rash, skin peeling, and/or opening of a healed wound. It may cause sudden, rapid loss of skin, which can result in fluid loss. Bevacizumab may cause weight loss and/or dehydration. It may cause low levels of potassium in the blood, which can lead to weakness. It may cause low levels of sodium in the blood, which can cause you to retain water. It may cause frequent urination, a strong desire to urinate immediately, and/or narrowing of the tube that carries urine from each kidney to the bladder.

Erlotinib hydrochloride may cause rash, dehydration, stomach pain, acne, and/or itchy skin. It may damage the kidneys (including kidney failure), ovaries, and/or hair follicles causing hair loss. It may cause headache, nosebleeds, and/or cough. It may cause abnormal results in liver function tests, which may mean liver damage. It may cause dry skin, dry lips and/or drying of the lining of the nose and/or mouth, with mouth pain and/or changes in taste.

Erlotinib hydrochloride may cause skin flushing, swelling, and/or skin sensitivity to sunlight. It may cause shedding, peeling, and/or scaling off of skin, drying of salivary glands, and/or inflammation of mucous membranes. It may cause dry eyes, decreased vision, blurred vision, and/or corneal damage, which may be permanent. It may cause vaginal bleeding and/or breakdown of the lining of the womb. It may cause fibrous tissue in the lungs, ulcers, black tarry stools, and/or vomit made up largely of blood. It may cause fever, weakness, indigestion, constipation or diarrhea, and/or changes in blood pressure and/or weight. It may cause loss of appetite, nausea, and/or vomiting. It may cause confusion, depression, agitation, irritability,

and/or muscle, joint, and/or bone aches. Erlotinib hydrochloride may cause difficulty urinating, ringing in the ears, and/or dizziness. It may cause intestinal bleeding, gas, and/or decrease in blood flow to organs due to blockage of blood vessels. It may cause blood clots in veins and arteries. It may cause dry mouth, irritation of the chest, difficulty sleeping, and/or irritation of the stomach or bowels, which may lead to ulcers (lining breakdown) and/or bleeding.

Erlotinib hydrochloride may interact with warfarin (Coumadin®), a drug used to decrease blood clotting. You should inform your doctor if you are taking this drug. Erlotinib hydrochloride may also interact with certain other medications/substances. These include fluconazole (Diflucan), cimetidine (Tagamet), erythromycin, St. John's wort, and grapefruit juice. Because of these interactions, and others that are not listed here, participants need to tell the study doctor about all medications, herbal remedies, vitamins, and supplements they are taking.

Erlotinib hydrochloride may cause the immune system to react against the drug, which may cause breathing difficulty. The drug may cause decreased oxygen in the blood, rapid heartbeat, and/or high blood pressure, which may cause lung damage. The drug may cause a lung disorder called interstitial lung disease (ILD). It may also cause high white blood cell count and inflammation of the lungs, pancreas, and/or lining of the mouth.

Combining bevacizumab with chemotherapy and radiation may increase the risk of bowel perforation.

Blood draws may cause pain, bleeding, and/or bruising. You may faint and/or develop an infection with redness and irritation of the vein at the site where blood is drawn. Frequent blood collection may cause anemia (low red blood cell count), which may create a need for blood transfusions.

Questionnaires may contain questions that are sensitive in nature. You may refuse to answer any question that makes you feel uncomfortable. If you have concerns after completing the questionnaire, you are encouraged to contact the study doctor.

This research study may involve unpredictable risks to the participants.

Pregnancy Related Risks

4a. Because the treatment used in this study can affect an unborn or breastfeeding baby, you should not become pregnant, breastfeed a baby, or father a child while on this study. You must practice birth control during the study if you are sexually active. If you are pregnant, you will not be enrolled on this study.

Birth Control Specifications: Oral contraceptive, other hormonal contraceptive, intrauterine device, diaphragm, and/or condom should be used.

Optional Procedures: Blood draws may cause pain, bleeding, and/or bruising. You may faint and/or develop an infection with redness and irritation of the vein at the site where blood is drawn. Frequent blood collection may cause anemia (low red blood cell count), which may create a need for blood transfusions.

5. POTENTIAL BENEFITS

If you appear to be benefitting from the study drugs and radiation, the additional experimental therapy that you receive may help to control the disease. Future patients may benefit from what is learned. There may be no benefits for you in this study.

Optional Procedures: There are no benefits for the participants taking part in the optional procedures. Future patients may benefit from what is learned. This information may help doctors to learn more about the use of the drugs in cancer treatment.

6. ALTERNATE PROCEDURES OR TREATMENTS

You may choose not to take part in this study. You may choose to receive the standard of care, such as capecitabine or 5-fluorouracil (5-FU) with radiation. You may choose to receive gemcitabine or other chemotherapy alone. You may choose to receive other investigational therapy, if available. You may choose not to have treatment for cancer at all. In all cases, you will receive appropriate medical care, including treatment for pain and other symptoms of cancer.

Optional Procedures: You may take part in this study without taking part in the optional procedures.

I understand that the following statements about this study are true:

7. This study is supported by: Genentech.
8. As required by the M. D. Anderson conflict of interest policy, a faculty member may not serve as the study chair for a study or primary physician for a subject on a study if he, she, or a family member holds any equity interest in the company sponsoring the study or has received cash from the company in excess of $10,000 within any 12-month period in the last three years.

   Investigator Name(s) and Type(s) of Interest(s)
   Dr. Christopher Crane (Study Co-Chair) has received compensation from Genentech as a speaker/preceptor. The amount recieved was within the limits of the conflict of interest policy.

9. In some instances of medical emergency, it is possible that I may be cared for by a physician and/or administrator who has some form of an equity, stock option, or other interest in the sponsor or supporter of this study. If I want to receive updated information regarding the financial interests of any physician or M. D. Anderson personnel who has cared for me, I may call the Conflict of Interest Coordinator at 713-792-3220. Upon request, I will be given access to information that will let me know if the UT System or M. D. Anderson has a conflict of interest, and I will be given the names of all physicians, administrators, and/or M. D. Anderson personnel who have a financial interest in Genentech.

10. My participation in this research study is strictly voluntary.

11. I may ask any questions I have about this study, including financial considerations, of the study chair. I may contact the study chair, Dr. Sunil Krishnan, at 713-563-2361. I may also contact the Chairman of M. D. Anderson's Institutional Review Board (IRB) at 713-792-2933 with any questions that have to do with this study or my rights as a study participant.

12. I may refuse to participate in this study without any penalty or loss of benefits to which I am otherwise entitled. I may also withdraw from participation in this study at any time without any penalty or loss of benefits. I should first discuss leaving the study with my physician. If I withdraw from this study, I may still be treated at M. D. Anderson.

13. I understand that the study may be changed or stopped at any time by the study chair, the IRB of M. D. Anderson, or Genentech.

14. I will be informed of any new findings that might affect my willingness to continue participating in the study.

15. M. D. Anderson will take appropriate steps to keep my personal information private. However, there is no guarantee of absolute privacy. The U.S. Food and Drug Administration (FDA), the IRB of M. D. Anderson, and/or Genentech might review my record to collect data or to see that the research is being done safely and correctly. Under certain circumstances, the FDA could be required to reveal the names of participants.

16. If I suffer injury as a direct result of participation in this study, M. D. Anderson will provide medical care. However, this medical care will be billed to my insurance or me in the ordinary manner. I understand that I will not be offered reimbursement of expenses or financial compensation from M. D. Anderson or Genentech for this injury. I may also contact the Chairman of M. D. Anderson’s IRB at 713-792-2933 with questions about study-related injuries.

17. Certain tests, procedures, and/or medications that I may receive as part of this study may be free to me because they are for research purposes only. However, my insurer or I may be financially responsible for the cost of supportive care and treatment of any complications resulting from the research tests, procedures, and/or medications, such as hospitalization, nausea, vomiting, low blood cell counts, and

dehydration. Standard medical care that I receive under this research study will be billed to my insurer and/or me in the ordinary manner. I should learn before participating in this study which part of the research-related care will be free, which costs my insurer will pay for, and which costs will be my responsibility.

18. I understand that there are no plans to provide any compensation to me for any patents or discoveries that may result from my participation in this research. I will receive no payment for taking part in this study.

Birth Control
I understand that I must practice birth control. Female participants should not breast-feed while on study. If I become pregnant, or suspect that I am pregnant, I must notify my physician immediately. Getting pregnant will result in removal from participation in this study.

Authorization for Use and Disclosure of Protected Health Information:

A. During the course of this study, the research team at M. D. Anderson will be collecting information about you that they may share with the FDA and/or Genentech. This information may include your medical history, treatment schedule, and the results of any of your tests, therapies, or procedures. The purpose of collecting and sharing this information is to learn about how the treatment affects the disease and any side effects you may experience as a result of your treatment.

Your doctor and the research team may share study information with certain individuals. These individuals may include representatives of the FDA, Genentech, clinical study monitors who verify the accuracy of the information, individuals with medical backgrounds who determine the effect that the treatment has on the disease, and/or individuals who put all the study information together in report form. The M. D. Anderson research team may provide this information to the FDA, and/or Genentech at any time.

You have the right to see and reproduce your records related to the research study for as long as this information is held by the study chair or M. D. Anderson. However, in some studies, in order to ensure the scientific value of the study, participants are not able to view or reproduce their study records until the research has been completed with all participants in the study. If possible for this study, your doctor will be able to discuss your clinical test results with you.

B. There is no expiration date for the use of this information as stated in this authorization. You may withdraw your authorization to share your personal health information at any time in writing. Instructions on how to do this can be found in the M. D. Anderson Notice of Privacy Practices (NPP). You may contact the Office of Protocol Research at 713-792-2933 with questions about how to find the NPP. If you withdraw your authorization, you will be removed from the study, and the study chair and staff will no longer use or disclose your personal health information in connection with this study, unless the study chair or staff needs to use or disclose some of your research-related personal health information to preserve the scientific value of the study. M. D. Anderson, and/or Genentech may use any study data that were collected before you canceled your authorization.

C. If you refuse to provide your authorization to disclose your protected health information, you will not be able to participate in this research project.

D. Your personal health information will be protected according to state and federal law. However, there is no guarantee that your information will remain confidential, and it may be re-disclosed at some point.

Please Do Not Use for Patient Consent

Go to the PDOL Homepage to access the Informed Consent Printer Database

CONSENT/AUTHORIZATION FOR TREATMENT AND OPTIONAL PROCEDURES

(Mark choice with an "X")

I elect to ____ or not to ____ have additional blood drawn to learn if bevacizumab is having an effect on the tumor and to learn the type of effect it may be having, as an optional procedure.

Patient's Initials ________

Having read and understood the above and having had the chance to ask questions about this study and reflect and consult with others as needed, I give the study chair permission to enroll me on this study. By signing this consent form, I am not giving up any of my legal rights. I have been given a signed copy of this consent.

SAMPLE -- NOT FOR USE IN CONSENTING PATIENTS

SIGNATURE OF PARTICIPANT DATE

I was present during the explanation of the research to be performed under Protocol **2007-0044**.

SAMPLE -- NOT FOR USE IN CONSENTING PATIENTS

SIGNATURE OF WITNESS OTHER THAN PHYSICIAN OR STUDY CHAIR TO THE VERBAL CONSENT PRESENTATION DATE

SAMPLE -- NOT FOR USE IN CONSENTING PATIENTS

SIGNATURE OF PERSON RESPONSIBLE & RELATIONSHIP DATE

I have discussed this clinical research study with the participant and/or his or her authorized representative, using a language that is understandable and appropriate. I believe that I have fully informed this participant of the nature of this study and its possible benefits and risks and that the participant understood this explanation.

SAMPLE -- NOT FOR USE IN CONSENTING PATIENTS

SIGNATURE OF STUDY CHAIR OR PERSON OBTAINING CONSENT DATE

Translator

I have translated the above informed consent into ____________________ and assisted the study chair in the consenting process (Name of Language) for this participant.

SAMPLE -- NOT FOR USE IN CONSENTING PATIENTS

NAME OF TRANSLATOR	SIGNATURE OF TRANSLATOR	DATE
