# Supplementary material for: Phase I Trial of Consolidative Radiotherapy with Concurrent Bevacizumab, Erlotinib and Capecitabine for Unresectable Pancreatic Cancer
Source: PLoS One. 2016 Jun 23;11(6):e0156910. doi: 10.1371/journal.pone.0156910 (PMC4919049; doi:10.1371/journal.pone.0156910)
Supplement: S3 File — (PDF) [file pone.0156910.s003.pdf]

THE UNIVERSITY OF TEXAS
MD Anderson Cancer Center

Protocol Page

Phase I Trial of radiotherapy with concurrent bevacizumab, erlotinib and capecitabine for locally advanced pancreatic cancer
2007-0044

Core Protocol Information

**Short Title**	Pancreas bevacizumab, erlonitib, capecitabine, radiation
**Study Chair:**	Sunil Krishnan
**Additional Contact:**	Benson T. Mathai<br>Toni Williams<br>Victoria Cox
**Department:**	Radiation Oncology
**Phone:**	713-563-2361
**Unit:**	097
**Full Title:**	Phase I Trial of radiotherapy with concurrent bevacizumab, erlotinib and capecitabine for locally advanced pancreatic cancer
**Protocol Type:**	Standard Protocol
**Protocol Phase:**	Phase I
**Version Status:**	Activated -- Closed to new patient entry as of 05/24/2016
**Version:**	16
**Submitted by:**	Victoria Cox--5/19/2016 8:12:10 AM
**OPR Action:**	Accepted by: Debbie D. Stroughter -- 5/19/2016 3:46:08 PM

Which Committee will review this protocol?

(•) The Clinical Research Committee - (CRC)

Protocol Body

1.0 Background

1.1 PANCREATIC CANCER

Pancreatic cancer is the third most common gastrointestinal malignancy and the fifth leading cause of cancer-related death in western countries. Unfortunately, less than 10% of patients are eligible for a margin-negative surgical resection, the only potentially curative treatment for pancreatic cancer, which confers a 15-25% rate of 5-year overall survival (OS) [1]. About two-thirds of all pancreatic cancer patients have radiographically detectable metastatic disease at presentation and the remaining patients have locally advanced unresectable disease. Typically, standard treatment for locally advanced pancreatic cancer consists of a combination of chemotherapy and radiation therapy in the United States, but the integration of these modalities and the respective dose schedules vary considerably.

Nearly 20 years ago, external-beam radiation therapy (EBRT) and concomitant 5-fluorouracil (5-FU) chemotherapy (chemoradiation) was shown to prolong survival compared to radiation alone in patients with locally advanced adenocarcinoma of the pancreas in a landmark randomized trial conducted by the GITSG (Gastrointestinal Tumor Study Group). Patients who received 40 Gy and 60 Gy with bolus 5-FU survived a median of 36 weeks (p=0.02) and 40 weeks (p=0.01) respectively, which was a statistically longer duration than radiation alone (median of 20 weeks) [2]. In addition to being shown to be a better treatment than radiotherapy alone, concurrent chemoradiation has been shown to be superior to chemotherapy alone [2, 3]. Patients were randomized to receive streptozocin, mitomycin, and 5-fluorouracil vs. the same chemotherapy followed by 5-FU based chemoradiation. Patients receiving the sequence of chemotherapy followed by chemoradiation were shown to have a significantly longer one-year overall survival (41% vs 19%, p = 0.02) [3]. Both radiographic local and distant progression occur in 60-70% of patients with locally advanced disease treated with chemoradiation. In the Phase II RTOG 98-12 study, 132 patients with unresectable cancer of the pancreas were treated with the combination of weekly paclitaxel (50mg/m2) and external beam irradiation (50.4 Gy in 28 fractions over 5.5 weeks). The median survival was noted to be 11.2 months (95% CI 10.1, 12.3) with estimated 1-and 2-year survivals of 43% and 13%, respectively [4]. This median survival is better than historical results achieved with irradiation and fluoropyrimidines, and serves as the benchmark for statistical comparison within the Radiation Therapy Oncology Group for locally advanced pancreatic cancer.

Pancreatic cancer has a very high rate of both local and distant disease progression. Full-dose systemic therapy followed sequentially by a well-tolerated chemoradiation regimen takes

advantage of the most effective treatments and effectively addresses the pattern of disease recurrence. Since the survival of patients with locally advanced pancreatic cancer is typically measured in months, and patients have significant disease-related nutritional challenges, chemotherapy and chemoradiation regimens should be well tolerated. It is clear that gemcitabine-based systemic therapy not only is well tolerated, but is also has a modest survival benefit over 5-fluorouracil [5]. However, the therapeutic index for concurrent gemcitabine-based chemoradiation is very narrow and may not permit addition of other agents [6]. Thus, concurrent 5-FU remains the standard when used with radiation in pancreatic cancer because it has comparable efficacy and less toxicity than gemcitabine. Capecitabine is a novel fluoropyrimidine designed for oral administration that mimics protracted venous infusions of 5-FU and is more convenient and less toxic to administer with radiation. It is converted to the cytotoxic agent fluorouracil through a series of enzymatic steps *in vivo*. The final step in conversion to fluorouracil is by thymidine phosphorylase, which is found in higher levels in tumor cells than in normal tissues. This increased rate of conversion to the active cytotoxic agent at the tumor site minimizes the exposure of normal body tissues to systemic 5-FU. Capecitabine has been shown to have a superior toxicity profile than 5-FU in both the metastatic and adjuvant settings in patients with colorectal cancer when compared to 5-FU/LV [7, 8]. At The University of Texas M. D. Anderson Cancer Center, capecitabine–based chemoradiotherapy delivered to the gross tumor alone has been extremely well tolerated with less than 5% of patients experiencing grade 3 gastrointestinal toxicity and has had significant local tumor activity. When capecitabine was administered continuously (7 days per week) throughout a 38-day course of radiotherapy (50.4 Gy in 28 fractions), therapy was extremely well tolerated at the recommended dose of 825 mg/m2 PO BID. The dose limiting toxicity was hand foot syndrome, and there were no grade 3 gastrointestinal adverse events. So it appears that capecitabine and radiation have independent (mutually exclusive) toxicities.

Efforts to improve on this regimen of chemoradiation therapy have focused on cytostatic agents that are well tolerated and having specific mechanisms of action directed against specific pathways that are relevant to the biology of pancreatic cancer. Patients with pancreatic cancer are challenged by nutritional deficiency secondary to pancreatic exocrine deficiency and anorexia. Therefore, a well-tolerated cytotoxic backbone is critical in trials evaluating novel targeted agents in pancreatic cancer.

Our goal is to explore the tolerability of the addition of erlotinib to the current regimen (see Section 1.2.1) of bevacizumab and capecitabine-based chemoradiation for locally advanced unresectable pancreatic cancer, and to look for preliminary evidence of an increase in the response rate. Increasing the response rate may increase rates of margin-negative resection in

these patients. Furthermore, improvements in neoadjuvant therapy in these patients could potentially increase local control and disease-free survival.

1.2 Bevacizumab CLINICAL Experience

1.2.1 Bevacizumab Clinical Trials

Bevacizumab, a monoclonal antibody directed against vascular endothelial growth factor (VEGF), has been studied in a multitude of Phase I, II, and III clinical trials in more than 5000 patients and in multiple tumor types. The following discussion summarizes bevacizumab's safety profile and presents some of the efficacy results pertinent to this particular trial. Please refer to the bevacizumab Investigator Brochure for descriptions of all completed Phase I, II, and III trials reported to date.

In a large phase III study (AVF2107g) in patients with metastatic colorectal cancer, the addition of bevacizumab, a monoclonal antibody directed against vascular endothelial growth factor (VEGF), to irinotecan/5-fluorouracil/leucovorin (IFL) chemotherapy resulted in a clinically and statistically significant increase in duration of survival, with a hazard ratio of death of 0.660 (median survival 15.6 vs. 20.3 months; $p < 0.0001$) [9]. Similar increases were seen in progression-free survival (6.2 vs. 10.6 months; $p < 0.0001$), overall response rate (35% vs. 45%; $p < 0.0029$), and duration of response (7.1 vs. 10.4 months; $p < 0.0014$) for the combination arm versus the chemotherapy only arm. In addition, bevacizumab has been shown to increase survival when added to 5-FU and leucovorin in patients with metastatic colorectal cancer [10].

Based on the survival advantage demonstrated in Study AVF2017g, bevacizumab was designated for priority review and was approved on 26 February 2004 in the United States for the first-line treatment in combination with IV 5-FU-based chemotherapy for subjects with metastatic colorectal cancer. It has since also been approved for the second-line treatment of patients with metastatic colorectal cancer and (in combination with carboplatin and paclitaxel) for the first-line treatment of patients with unresectable, locally advanced, recurrent or metastatic non-squamous, non-small cell lung cancer.

Bevacizumab may also play an important role in the neoadjuvant treatment of locally advanced rectal cancers. Willett et al. are investigating the addition of bevacizumab to 5-FU for the pre-operative treatment of rectal cancer patients [11]. Bevacizumab appears to decrease tumor perfusion, vascular volume and microvascular density in rectal adenocarcinomas [11]. At The University of Texas M.D. Anderson Cancer Center, concurrent capecitabine and radiotherapy has

been used in locally advanced pancreatic cancer in a phase I trial of 48 patients evaluating chemoradiation with concurrent bevacizumab. Using 825 mg/m^2/day (7 day schedule), there has been less than 5% G3 GI toxicity. In a subsequent, phase II trial, the recommended dose of capecitabine was 825 mg/m^2/day on a 5-day schedule (Monday-Friday on days of radiotherapy). There were 9 confirmed partial responses and 4 patients who were deemed unresectable initially that underwent margin-negative resections after this treatment [12]. That study also concluded that concurrent bevacizumab did not significantly increase the acute toxicity of a relatively well-tolerated chemoradiotherapy regimen of capecitabine and radiation therapy. These results have spurred continuing interest in this regimen and multiple groups including the Radiation Therapy Oncology Group (RTOG) and American College of Surgeons Oncology Group (ACOSOG) are exploring similar combinations of agents.

CALGB 80303, a phase III multi-center randomized trial that evaluated bevacizumab for the first-line treatment of metastatic pancreatic cancer, was stopped early because it did not meet its primary endpoint of overall survival. In this study, 602 patients were randomized to receive gemcitabine + bevacizumab or gemcitabine + placebo. A pre-specified futility analysis indicated that it is very unlikely that significant differences in overall survival will be shown between treatment arms as the data mature. The study was not stopped due to safety events and no new safety concerns related to bevacizumab were observed in this trial. The negative results of the CALGB study may be limited to metastatic pancreatic cancer patients.

We have recently retrospectively compared treatment outcomes for locally advanced pancreatic cancer in 47 patients treated with bevacizumab to that of a large historical cohort of 323 patients treated with chemoradiation with or without induction chemotherapy. On univariate analysis, the addition of bevacizumab to chemoradiation appeared to improve overall survival compared to historical regimens containing fluoropyrimidines or gemcitabine alone (15.0 months compared to 9 months). The magnitude of this difference was clinically significant, but did not hold up on multivariate analysis, possibly due to the relatively small numbers in this group. Results of this analysis were presented at ASTRO 2006 and are being written up as a manuscript.

a. Safety Profile

In the initial Phase I and II clinical trials, four potential bevacizumab-associated safety signals were identified: hypertension, proteinuria, thromboembolic events, and hemorrhage. Additional completed Phase II and Phase III studies of bevacizumab as well as spontaneous reports have further defined the safety profile of this agent. Bevacizumab-associated adverse events identified in phase III trials include congestive heart failure (CHF), gastrointestinal perforations, wound healing complications, and arterial thromboembolic events (ATE). These and other safety signals are described in further detail as follows and in the bevacizumab Investigator Brochure.

Hypertension: Hypertension has been commonly seen in bevacizumab clinical trials to date and oral medications have been used to manage the hypertension when indicated. Grade 4 and 5 hypertensive events are rare. Clinical sequelae of hypertension are rare but have included hypertensive crisis, hypertensive encephalopathy, and reversible posterior leukoencephalopathy syndrome (RPLS) [13, 14]. RPLS may include signs and symptoms of headache, altered mental function, seizures, and visual disturbances / cortical blindness and requires treatment, which should include control of hypertension, management of specific symptoms, and discontinuation of bevacizumab.

Proteinuria: Proteinuria has been commonly seen in bevacizumab clinical trials to date. The severity of proteinuria has ranged from asymptomatic and transient events detected on routine dipstick urinalysis to nephrotic syndrome; the majority of proteinuria events have been grade 1 or 2. In study AVF2107g, none of the 118 patients receiving bolus-IFL plus placebo, three of 158 patients (2%) receiving bolus-IFL plus bevacizumab, and two of 50 (4%) patients receiving 5-FU/LV plus bevacizumab who had a 24-hour collection experienced grade 3 proteinuria (> 3.5 g protein/24 hr). Rare events of nephrotic syndrome have occurred, and bevacizumab should be discontinued in patients with nephrotic syndrome.

Thromboembolic Events: Both venous and arterial thromboembolic (TE) events, ranging in severity from catheter-associated phlebitis to fatal, have been reported in patients treated with bevacizumab in the colorectal cancer trials and, to a lesser extent, in patients treated with bevacizumab in NSCLC and breast cancer trials. In the phase III pivotal trial in metastatic CRC, there was a slightly higher rate of **venous TE** events that was not statistically significant in patients treated with bevacizumab plus chemotherapy compared with chemotherapy alone (19% vs. 16%). There was also a higher rate of **arterial TE** events (3% vs. 1%) such as myocardial infarction, transient ischemia attack, cerebrovascular accident/stroke and angina/unstable angina ***.*** A pooled analysis of the rate of arterial TE events from 5 randomized studies (1745 patients) showed that treatment with chemotherapy plus bevacizumab increased the risk of having an

arterial TE event compared with chemotherapy alone (3.8% vs. 1.7%, respectively) [15]. Furthermore, subjects with certain baseline characteristics (age $\geq$ 65 years and/or a history of a prior arterial TE event) may be at higher risk of experiencing such an event. . See the bevacizumab Investigator Brochure for additional information on risk factors.

Aspirin is a standard therapy for primary and secondary prophylaxis of arterial thromboembolic events in patients at high risk of such events, and the use of aspirin $\leq$ 325 mg daily was allowed in the five randomized studies discussed above. Use of aspirin was assessed routinely as a baseline or concomitant medication in these trials, though safety analyses specifically regarding aspirin use were not preplanned. Due to the relatively small numbers of aspirin users and arterial thromboembolic events, retrospective analyses of the ability of aspirin to affect the risk of such events were inconclusive. However, similarly retrospective analyses suggested that the use of up to 325 mg of aspirin daily does not increase the risk of grade 1-2 or grade 3-4 bleeding events, and similar data with respect to metastatic colorectal cancer patients were presented at ASCO 2005 [16]. Further analyses of the effects of concomitant use of bevacizumab and aspirin in colorectal and other tumor types are ongoing.

Gastrointestinal perforation Patients with metastatic carcinoma may be at increased risk for the development of gastrointestinal perforation when treated with bevacizumab and chemotherapy. Bevacizumab should be permanently discontinued in patients who develop gastrointestinal perforation. A causal association of intra-abdominal inflammatory process and gastrointestinal perforation to bevacizumab has not been established. Nevertheless, caution should be exercised when treating patients with intra-abdominal inflammatory processes with bevacizumab. Gastrointestinal perforation has been reported in other trials in non-colorectal cancer populations (e.g., ovarian, renal cell, pancreas, and breast) and may be higher in incidence in some tumor types.

Wound healing complications: Wound healing complications such as wound dehiscence have been reported in patients receiving bevacizumab. In an analysis of pooled data from two trials in metastatic colorectal cancer, patients undergoing surgery 28-60 days before study treatment with 5-FU/LV plus bevacizumab did not appear to have an increased risk of wound healing complications compared to those treated with chemotherapy alone [17]. Surgery in patients currently receiving bevacizumab is not recommended. No definitive data are available to define a safe interval after bevacizumab exposure with respect to wound healing risk in patients receiving elective surgery; however, the estimated half life of bevacizumab is 20 days. Bevacizumab should be discontinued in patients with severe wound healing complications.

Hemorrhage: Overall, grade 3 and 4 bleeding events were observed in 4.0% of 1132 patients treated with bevacizumab in a pooled database from eight phase I, II, and III clinical trials in multiple tumor types[18]. The hemorrhagic events that have been observed in bevacizumab clinical studies were predominantly tumor-associated hemorrhage (see below) and minor mucocutaneous hemorrhage.

Tumor-associated hemorrhage – was observed in phase I and phase II bevacizumab studies. Six serious events, of which 4 had fatal outcome, were observed in a phase II trial of patients with non-small cell lung cancer receiving bevacizumab. These events occurred suddenly and presented as major or massive hemoptysis in patients with either squamous cell histology and/or tumors located in the center of the chest in close proximity to major blood vessels. In five of these cases, these hemorrhages were preceded by cavitation and/or necrosis of the tumor. Tumor-associated hemorrhage was also seen rarely in other tumor types and locations, including central nervous system (CNS) bleeding in a patient with hepatoma with occult CNS metastases and continuous oozing of blood from a thigh sarcoma with necrosis.

Across all bevacizumab clinical trials, mucocutaneous hemorrhage has been seen in 20%-40% of patients treated with bevacizumab. These were most commonly grade 1 epistaxis that lasted less than 5 minutes, resolved without medical intervention and did not require any changes in bevacizumab treatment regimen. There have also been less common events of minor mucocutaneous hemorrhage in other locations, such as gingival bleeding and vaginal bleeding.

Congestive heart failure: CHF has been reported in bevacizumab clinical trials and may be increased in incidence in patients with prior exposure to anthracyclines or prior irradiation to the chest wall. In a phase III trial (AVF2119g) of capecitabine with or without bevacizumab for metastatic breast cancer, 7 subjects (3.1%) who received capecitabine plus bevacizumab developed clinically significant CHF compared with 2 subjects (0.9%) treated with capecitabine alone; of note, all subjects in this trial had had prior anthracycline treatment. In addition, 2 subjects had a left ventricular ejection fraction < 50% at baseline and 2 others had prior left chest wall irradiation. A recently published phase II study in subjects with refractory acute myelogenous leukemia reported 5 cases of cardiac dysfunction (CHF or decreases to <40% in left ventricular ejection fraction) of 48 subjects treated with sequential cytarabine, mitoxantrone, and bevacizumab. All but one of these subjects had significant prior exposure to anthracyclines as well[19]. Other studies are ongoing in this patient population. Patients receiving anthracyclines or with prior exposure to anthracyclines should have a baseline MUGA or ECHO with a normal ejection fraction.

Additional Safety Signals: Other safety concerns seen to date—asthenia, pain, headache, fever, chills, rash, infection, epistaxis, and mouth ulceration—are not thought to be clinically significant in that they rarely or never required treatment or study drug discontinuation. Additional Adverse Events: See the bevacizumab Investigator Brochure for additional details regarding the safety experience with bevacizumab.

1.3 ERLOTINIB

1.3.1 Description

Erlotinib is an orally administered Human Epidermal Growth Factor Receptor Type 1/Epidermal Growth Factor Receptor (HER1/EGFR) tyrosine kinase inhibitor. The chemical name for erlotinib is N-(3-ethynylphenyl)-6,7-bis(2-methoxyethoxy)- 4-quinazolinamine. Erlotinib has the following structural formula:

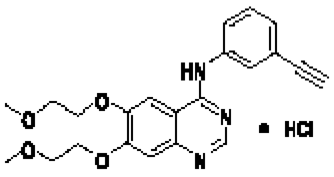

1.3.2 Mechanism of Action

Erlotinib inhibits the intracellular phosphorylation of epidermal growth factor receptor (EGFR)-associated tyrosine kinase. EGFR is expressed on the cell surface of normal cells and cancer cells.

1.3.3 Erlotinib Clinical Experience

To date, erlotinib has been studied clinically in more than 4000 healthy patients and patients (excluding patients exposed to placebo) in a number of Phase I, II, and III studies.

a. Dose Selection for Single-Agent Trials of Erlotinib

Phase I trials of erlotinib explored both schedule and dose to evaluate the safety, tolerability, and pharmacokinetic profile of the compound given as a single agent. A number of pharmacokinetic trials in healthy patients have been conducted, along with three classic Phase I trials in patients with advanced cancer. The single-agent maximum tolerated dose (MTD) was estimated to be 150 mg administered once daily.

The primary toxicities of single-agent erlotinib consisted of rash (dermatosis), diarrhea, nausea,

The primary toxicities of single-agent erlotinib consisted of rash (dermatosis), diarrhea, nausea, fatigue, stomatitis, vomiting, and headache. When given daily, dose-limiting toxicity (diarrhea) was observed at 200 mg/day. At 150 mg/day, diarrhea was manageable with the addition of loperamide therapy; this dose was considered the maximal tolerated dose.

Rash (variously referred to as dermatitis, acneiform rash, or maculopapular rash) has been variable in onset, duration, and severity, but typically appears on the face, neck, scalp, chest, and back starting after ~1 week of treatment. The mechanistic basis of the rash remains uncertain; histopathologic examination of biopsies of the rash demonstrated inflammatory cell infiltrate and mild epidermal hyperproliferation. In some cases, the rash gradually improved despite continued dosing and, in general, resolved without sequelae following erlotinib discontinuation. The rash did not result in study discontinuation in patients with cancer in the Phase I trials.

Laboratory abnormalities observed infrequently with single-agent erlotinib involved primarily liver function tests, including elevation of ALT, AST, and/or bilirubin.

Selection of the 150 mg/day dose of erlotinib for subsequent single-agent studies was based on pharmacokinetic parameters, as well as the safety and tolerability profile of this dose in Phase I trials in heavily pretreated patients with advanced cancer. Drug levels seen in patients with cancer receiving the 150 mg/day dose were consistently above the average plasma concentration of 500 ng/mL targeted for clinical efficacy.

b. Pharmacokinetics

Oral erlotinib is well absorbed and has an extended absorption phase, with mean peak plasma levels occurring at 3 hours after oral dosing of 150 mg/dL at steady state. A study in healthy patients provided an estimate of bioavailability of 59% (95% CI: 55%, 63%). The time to reach steady-state plasma concentration was ~5 days. The accumulation ratio with daily dosing of Erlotinib was estimated to be 2.0. From a population pharmacokinetic analysis of 708 patients, the median trough concentration (C_{min}) 24 hours following the previous dose was 1041 (697) ng/mL. Median AUC achieved during the dosing interval at steady state was 19,801 ng hr/mL. Exposure after an oral dose is increased by food.

There is extensive binding of erlotinib and metabolites to both serum albumin and AAG (alpha-1-acid glycoprotein), with total plasma protein binding for erlotinib and OSI-420 of ~95% and 91%, respectively. Erlotinib is extensively metabolized in the liver by the hepatic cytochromes in humans—primarily by CYP3A4 and to a lesser extent by CYP1A2. The primary metabolite of erlotinib, OSI-420, has potency comparable to that of erlotinib, but is present at levels that are 10% of erlotinib levels. Erlotinib is excreted predominantly via the feces (90%).

levels that are 10% of erlotinib levels. Erlotinib is excreted predominantly via the feces (90%).

The elimination half-life after a 150-mg oral dose is ~30 hours. In population-based data analyses, no relationships were identified between predicted steady-state trough concentration and patient age, body weight, sex, ethnicity, or creatinine clearance.

d. Phase II and III Trials in Patients with Advanced Cancer

Multiple Phase II trials evaluating the safety, tolerability, and antitumor activity of erlotinib have been conducted in patients with advanced, refractory malignancies including cancer of the head and neck, lung, aerodigestive tract, ovary, breast, central nervous system (glioma), and others. Erlotinib has been evaluated both as a single agent and administered concurrently with conventional chemotherapy agents using various doses and schedules.

Evidence of activity has been observed in squamous cell carcinoma of the head and neck, ovarian, breast and pancreatic carcinoma, non–small cell lung cancer (NSCLC), and glioblastoma multiforme (GBM). Patients received 150 mg/day of erlotinib in all of these studies except the GBM study where dose escalation was allowed until limited by rash and where a higher starting dose was tested in patients receiving concomitant enzyme inducing anti-epileptic drugs. Dose reduction was allowed in all studies in the case of intolerance. Diarrhea was treated with loperamide therapy and/or dose reduction. Rash was treated with a variety of agents, including oral and topical antibiotics, corticosteroids, and other agents.

Patients receiving erlotinib in combination with various chemotherapy agents have generally experienced the same type of adverse events (AEs) as with either agent alone.

The first randomized placebo controlled trial to demonstrate a survival advantage for an EGFR inhibitor was the Phase III study, BR21. This international trial, conducted by the National Cancer Institute of Canada Clinical Trial Group (NCIC CTG), included 731 patients with incurable Stage IIIB/IV NSCLC who have failed standard therapy for advanced or metastatic disease. Patients randomized in a 2:1 ratio to single-agent erlotinib 150 mg/day obtained a 42.5% improvement in median survival over placebo, from 4.7 to 6.7 months. The one-year survival increased significantly (from 22% to 31%) as did the median and 6 month PFS, response rate, and the time to deterioration of tumor related symptoms of pain, cough, and dyspnea [20].

In BR-21, of the 727 patients evaluable for safety (485 erlotinib, 242 placebo), the most common AEs in the erlotinib arm were rash (75% erlotinib, 17% placebo), diarrhea (54% erlotinib, 18% placebo) and stomatitis (17% erlotinib, 18% placebo) events. The majority of these events were mild to moderate in severity. The incidence of interstitial lung disease (ILD) reported was the same in the placebo and erlotinib groups at 0.8% in each arm.

Two large, Phase III, randomized studies in first-line NSCLC patients evaluated erlotinib in combination with platinum-based two-drug combination chemotherapy. A total of 1079 previously untreated patients received carboplatin/paclitaxel with either erlotinib or placebo in the TRIBUTE trial (OSI2298g) conducted in the United States [21]. An additional 1172 patients received cisplatin/gemcitabine plus either erlotinib or placebo in the TALENT trial (BO16411) conducted in 27 countries in Europe and other ex-U.S. locations (Gatzemeier U. et al.- Talent trial - ASCO 2004 - Abstract 7010). Neither study met its primary endpoint of improved overall survival or a secondary endpoint of improved time to disease progression or overall response rate. Overall, the number of adverse events and serious adverse events were well balanced between the two arms of each study, with two exceptions. As expected, rash and diarrhea occurred more frequently in the erlotinib arms. In the TRIBUTE study, more serious adverse events resulting in death were seen in the erlotinib arm compared with the placebo arm (53 vs. 27). Most of the apparent imbalance was due to events reported as pneumonia or progression of underlying cancer.

e. Pancreatic cancer

A randomized double-blind study was performed in 569 patients with locally advanced, unresectable or metastatic pancreatic cancer [22]. Patients were randomized to receive either erlotinib (100 mg once daily [n=261] or 150 mg once daily [n=24]) and gemcitabine, or placebo and gemcitabine, until disease progression or unacceptable toxicity. Results have been reported for patients receiving erlotinib at a dose of 100 mg once daily. Patients receiving erlotinib and gemcitabine had a significantly higher rate of overall survival compared to patients receiving placebo and gemcitabine (P=0.028). The median survival was 6.4 months in patients receiving erlotinib and gemcitabine, and 6.0 months in patients receiving placebo and gemcitabine. The effect of erlotinib appeared to be consistent across all patient subsets.

f. Patients with Hepatic or Renal impairment

The influence of hepatic metastases and/or hepatic dysfunction on the pharmacokinetics of erlotinib is not yet known. However, erlotinib is cleared predominately by the liver, and caution should be used when administering erlotinib to patients with hepatic dysfunction. erlotinib is also a strong inhibitor of the UDP-glucuronosyltransferase UGT1A1 enzyme responsible for the glucuronidation of bilirubin. Hyperbilirubinemia appears most often to be a side effect related to genetic polymorphisms of UGT1A1.

No clinical studies have been conducted in patients with compromised renal function since erlotinib and its metabolites are not significantly excreted by the kidneys.

1.3.4 Erlotinib Safety Profile

Pulmonary Toxicity

Serious interstitial lung disease-like events have been reported infrequently in patients receiving erlotinib. The overall incidence of erlotinib in nearly 5000 patients treated with erlotinib from all studies was approximately 0.7%.

Myocardial Infarction and Ischemia

Six patients (2.3%) receiving gemcitabine and erlotinib in the pancreatic cancer trial developed myocardial infarction/ischemia, with one death. In comparison, 3 patients (1.2%) receiving gemcitabine and placebo developed myocardial infarction/ischemia.

Cerebrovascular accident

Six patients (2.3%) receiving gemcitabine and erlotinib in the pancreatic cancer trial developed cerebrovascular accidents, with one death. In comparison, no patient receiving gemcitabine and placebo developed cerebrovascular accident.

Microangiopathic Hemolytic Anemia with Thrombocytopenia

Two patients (0.8%) receiving gemcitabine and erlotinib in the pancreatic cancer trial developed microangiopathic hemolytic anemia with thrombocytopenia. In comparison, no patient receiving gemcitabine and placebo developed microangiopathic hemolytic anemia with thrombocytopenia.

Liver Function Test Abnormalities

Liver-function test abnormalities have been observed in patients receiving single-agent erlotinib, but these were mostly transient or associated with liver metastases. Liver-function test abnormalities have been observed in patients receiving erlotinib and gemcitabine (11-14% grade 3-4).

Additional Safety Signals

Infrequent cases of gastrointestinal bleeding have reported with the use of erlotinib, some associated with concurrent NSAID or warfarin use. Infrequent cases of corneal ulcerations, and infrequent cases of grade 3 conjunctivitis and keratitis have been reported with the use of erlotinib.

1.3.5 Adverse Reactions

Rash

Rash was one of the most common adverse reaction in non-small cell lung cancer patients receiving single-agent erlotinib (9% grade 3-4) and in pancreatic cancer patients receiving erlotinib and gemcitabine (5% grade 3-4).

Diarrhea

Diarrhea was one of the most common adverse reaction in non-small cell lung cancer patients receiving single-agent erlotinib (6% grade 3-4) and in pancreatic cancer patients receiving erlotinib and gemcitabine (5% grade 3-4).

Other Adverse Reactions

Other common adverse reactions in pancreatic cancer patients receiving erlotinib and gemcitabine were fatigue, nausea (7% grade 3), and anorexia.

1.4 BEVACIZUMAB and Erlotinib Combination Studies

The strategy for combining therapeutic agents in cancer treatments has been successful in multiple tumor types, including NSCLC. A new series of clinical studies are now being designed and conducted to evaluate the combination of bevacizumab with erlotinib, particularly in NSCLC and RCC. This approach has scientific rationale because the two agents target different pathways involved in tumor growth and nonclinical studies in xenograft models have demonstrated that the combination of bevacizumab and erlotinib results in greater efficacy than either agent alone. Furthermore, because there is little to no overlap in toxicity profile between the two agents, the combination is expected to be well tolerated and may provide even greater benefit for patients who are unable to receive cytotoxic therapy.

An investigator-sponsored Phase I/II trial (OSI2486s) is currently being conducted to evaluate the combination of bevacizumab and erlotinib in subjects with relapsed or refractory NSCLC with non-squamous histology. This Phase I study evaluated three dose combinations to determine tolerability and pharmacokinetic profile of each agent when combined. Subjects whose disease had progressed following at least one chemotherapy regimen for advanced disease (Stage IIIb or IV) were treated with 100 mg/day erlotinib + 7.5 mg/kg bevacizumab every 3 weeks, 100 mg/day erlotinib + 15 mg/kg bevacizumab every 3 weeks, or 150 mg/day erlotinib + 15 mg/kg bevacizumab every 3 weeks. A total of 12 subjects were enrolled and treated (3, 3, and 6 per cohort, respectively). No dose-limiting toxicities were observed, and the pharmacokinetic profiles of both drugs did not appear to be affected by the combination.

For the Phase II portion of the study, 22 subjects are now evaluable for safety and efficacy,

For the Phase II portion of the study, 22 subjects are now evaluable for safety and efficacy, inclusive of the 12 subjects treated in Phase I. The majority of the subjects had a good performance status (11 with a Karnofsky score of 90%), adenocarcinoma histology (14 of 22 subjects), and fewer than two prior chemotherapy regimens (16 of 22). The most common adverse events were rash (20 of 22 subjects), diarrhea (14 of 22), proteinuria (11 of 22), and nausea (9 of 22). All of the events were Grade 1 or 2. There was one episode of Grade 1 hemoptysis and one episode of Grade 3 hypertension. Eleven of the 22 subjects (50%) experienced progressive disease after the first 6 weeks of therapy. Four partial responses (18.8%) and seven instances of stable disease (31.2%) were reported. Based on the preliminary data from this single-arm, Phase I/II study, it appears that the combination of bevacizumab and erlotinib is well tolerated by most subjects at maximum doses and has encouraging activity, supporting a larger, controlled study to assess the efficacy and tolerability of the regimen.

1.5 CAPECITABINE

1.5.1 Description

Capecitabine is a fluoropyrimidine carbamate with antineoplastic activity. It is an orally administered systemic prodrug of 5’-deoxy-5-fluorouridine (5’-DFUR) which is converted to 5-fluorouracil. The chemical name for capecitabine is 5’-deoxy-5-fluoro-N-[(pentyloxy) carbonyl]-cytidine and has a molecular weight of 359.35. Capecitabine has the following structural formula:

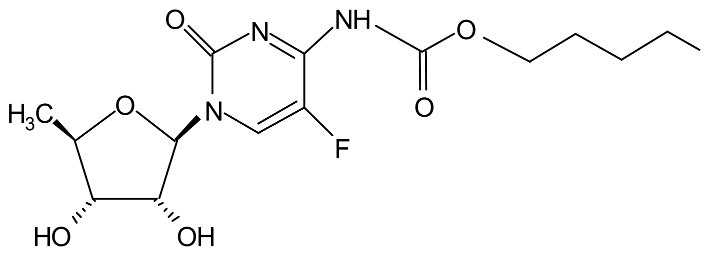

Capecitabine is a white to off-white crystalline powder with an aqueous solubility of 26 mg/mL at 20ºC. Capecitabine is supplied as biconvex, oblong film-coated tablets for oral administration. Each light peach-colored tablet contains 150 mg capecitabine and each peach-colored tablet contains 500 mg capecitabine. The inactive ingredients in capecitabine include: anhydrous lactose, croscarmellose sodium, hydroxypropyl methylcellulose, microcrystalline cellulose, magnesium stearate and purified water. The peach or light peach film coating contains hydroxypropyl methylcellulose, talc, titanium dioxide, and synthetic yellow and red iron oxides.

Drug Formulation, Packaging, and Storage

Capecitabine is commercially available as 150 mg or 500 mg tablets. Capecitabine tablets are packed in polyethylene bottles, containing either 120 x 150 mg tablets or 240 x 500 mg tablets. Capecitabine tablets should be stored at room temperature (15 to 30C) in the container in which they are provided.

1.5.2 Clinical Pharmacology

Capecitabine is relatively non-cytotoxic in vitro. This drug is enzymatically converted to 5-fluorouracil (5-FU) in vivo.

Bioactivation: Capecitabine is readily absorbed from the gastrointestinal tract. In the liver, a 60 kDa carboxylesterase hydrolyzes much of the compound to 5'-deoxy-5-fluorocytidine (5'-DFCR). Cytidine deaminase, an enzyme found in most tissues, including tumors, subsequently converts 5'-DFCR to 5'-deoxy-5-fluorouridine (5'-DFUR). The enzyme, thymidine phosphorylase (dThdPase), then hydrolyzes 5'-DFUR to the active drug 5-FU. Many tissues throughout the body express thymidine phosphorylase. Some human carcinomas express this enzyme in higher concentrations than surrounding normal tissues.

Metabolic Pathway of Capecitabine to 5-FU

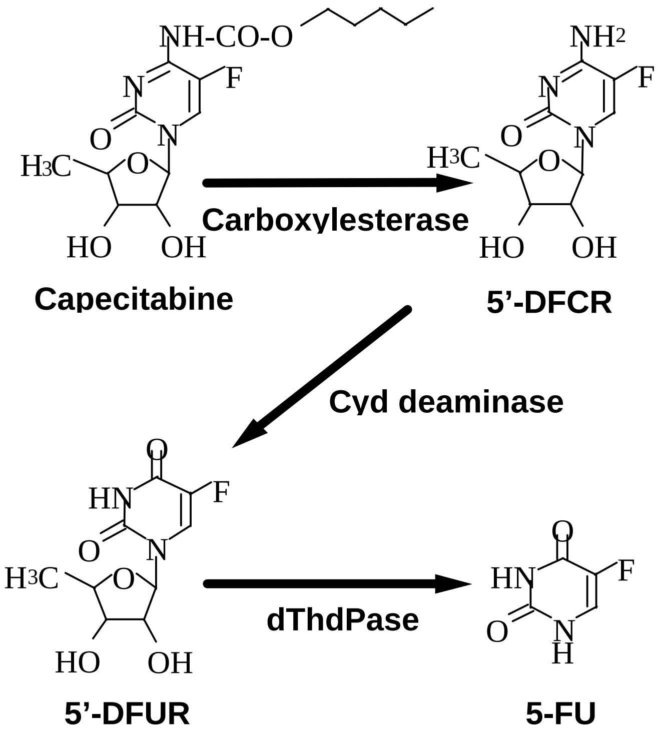

1.5.3 Mechanism of Action

Both normal and tumor cells metabolize 5-FU to 5-fluoro-2'-deoxyuridine monophosphate (FdUMP) and 5-fluorouridine triphosphate (FUTP). These metabolites cause cell injury by two different mechanisms. First, FdUMP and the folate cofactor, N5-10-methylenetetrahydrofolate, bind to thymidylate synthase (TS) to form a covalently bound ternary complex. This binding inhibits the formation of thymidylate from 2'-deoxyuridylate. Thymidylate is the necessary precursor of thymidine triphosphate, which is essential for the synthesis of DNA, so that a deficiency of this compound can inhibit cell division. Second, nuclear transcriptional enzymes can mistakenly incorporate FUTP in place of uridine triphosphate (UTP) during the synthesis of RNA. This metabolic error can interfere with RNA processing and protein synthesis.

1.5.4 Pharmacokinetics in Colorectal Tumors and Adjacent Healthy Tissue

Following oral administration of capecitabine 7 days before surgery in patients with colorectal cancer, the median ratio of 5-FU concentration in colorectal tumors to adjacent tissues was 2.9 (range from 0.9 to 8.0). These ratios have not been evaluated in breast cancer patients or compared to 5-FU infusion.

1.5.5 Human Pharmacokinetics

The pharmacokinetics of capecitabine and capecitabine metabolites have been evaluated in about 200 cancer patients over a dosage range of 500 to 3500 mg/m^2/day. Over this range, the pharmacokinetics of capecitabine and its metabolite, 5'-DFCR were dose proportional and did not change over time. The increases in the AUCs of 5'-DFUR and 5-FU, however, were greater than proportional to the increase in dose and the AUC of 5-FU was 34% higher on day 14 than on day 1. The elimination half-life of both parent capecitabine and 5-FU was about ¾ of an hour. The inter-patient variability in the C_{max} and AUC of 5-FU was greater than 85%.

1.5.6 Absorption, Distribution, Metabolism and Excretion

Capecitabine reached peak blood levels in about 1.5 hours (Tmax) with peak 5-FU levels occurring slightly later, at 2 hours. Food reduced both the rate and extent of absorption of capecitabine with mean Cmax and AUC decreased by 60% and 35%, respectively. The Cmax and AUC0- of 5-FU were also reduced by food by 43% and 21%, respectively. Food delayed Tmax of both parent and 5-FU by 1.5 hours. Plasma protein binding of capecitabine and its metabolites is less than 60% and is not concentration-dependent. Capecitabine was primarily bound to human albumin (approximately 35%). Capecitabine is extensively metabolized enzymatically to 5-FU. The enzyme dihydropyrimidine dehydrogenase hydrogenates 5-FU, the product of capecitabine metabolism, to the much less toxic 5-fluoro-5, 6-dihydro-fluorouracil (FUH2). Dihydropyrimidinase cleaves the pyrimidine ring to yield 5-fluoro-ureido-propionic acid (FUPA).

Finally, ureido-propionase cleaves FUPA to fluoro--alanine (FBAL) which is cleared in the urine. Capecitabine and its metabolites are predominantly excreted in urine; 95.5% of administered capecitabine dose is recovered in urine. Fecal excretion is minimal (2.6%). The major metabolite excreted in urine is FBAL, which represents 57% of the administered dose. About 3% of the administered dose is excreted in urine as unchanged drug.

1.5.7 Capecitabine Drug-Drug Interactions

Coumarin (Coumadin):

Drug interaction between capecitabine and coumarin anticoagulants has been reported. Altered coagulation parameters and/or bleeding have been reported in patients taking capecitabine concomitantly with coumarin-derivative anticoagulants such as warfarin and phenprocoumon. These events occurred within several days and up to several months after initiating capecitabine therapy and, in a few cases, within one month after stopping capecitabine and occurred in patients with and without liver metastases. In a drug interaction study with single dose warfarin administration, there was a significant increase in the mean AUC of S-warfarin. The maximum

administration, there was a significant increase in the mean AUC of S-warfarin. The maximum observed INR value increased by 91%. This interaction is probably due to an inhibition of cytochrome P450 2C9 by capecitabine and/or its metabolites.

Phenytoin:
Increased phenytoin plasma concentrations have been reported during concomitant use of capecitabine with phenytoin, suggesting a potential interaction. Patients taking phenytoin concomitantly with capecitabine should be regularly monitored (e.g. weekly phenytoin and albumin levels) for increased phenytoin plasma concentrations and associated clinical symptoms.

1.5.8 Clinical Studies

The recommended dose of capecitabine was determined in a open-label, randomized clinical study, exploring the efficacy and safety of continuous therapy with capecitabine (1331 mg/m^2/day in two divided doses, n=39), intermittent therapy with capecitabine (2510 mg/m^2/day in two divided doses, n=34), and intermittent therapy with capecitabine in combination with oral leucovorin (LV) (capecitabine 1657 mg/m^2/day in two divided doses, n=35; leucovorin 60 mg/day) in patients with advanced and/or metastatic colorectal carcinoma in the first-line metastatic setting. There was no apparent advantage in response rate to adding leucovorin to capecitabine; however, toxicity was increased. Capecitabine, 1250 mg/m^2 twice daily for 14 days followed by a 1-week rest, was selected for further clinical development based on the overall safety and efficacy profile of the three schedules studied.

Data from 2 open-label, multicenter, randomized, controlled clinical trials involving 1207 patients support the use of capecitabine in the first-line treatment of patients with metastatic colorectal carcinoma [7]. The two clinical studies were identical in design and were conducted in 120 centers in different countries. Study 1 was conducted in the US, Canada, Mexico, and Brazil; Study 2 was conducted in Europe, Israel, Australia, New Zealand, and Taiwan. Altogether, in both trials, 603 patients were randomized to treatment with capecitabine at a dose of 1250 mg/m^2 twice daily for 2 weeks followed by a 1-week rest period and given as 3-week cycles; 604 patients were randomized to treatment with 5-FU and leucovorin (20 mg/m^2 leucovorin IV followed by 425 mg/m^2 IV bolus 5-FU, on days 1 to 5, every 28 days).

Capecitabine was compared to bolus 5-FU and leucovorin in a randomized trial on adjuvant chemotherapy for stage III colon cancer patients. [8] Patients in the capecitabine arm had at least equivalent disease-free survival compared with patients in the 5-FU/leucovorin arm, with a trend towards superiority for capecitabine. Patients in the capecitabine arm also had a significantly

higher rate of relapse-free survival and a significantly lower rate of grade 3 or 4 toxicity.

1.5.9 Special Populations

A population analysis of pooled data from the two large controlled studies in patients with colorectal cancer (n=505) who were administered capecitabine at 1250 mg/m^2 twice a day indicated that gender (202 females and 303males) and race (455 white/caucasian patients, 22 black patients, and 28 patients of other race) have no influence on the pharmacokinetics of 5'-DFUR, 5-FU and FBAL. Age has no significant influence on the pharmacokinetics of 5'-DFUR and 5-FU over the range of 27 to 86 years. A 20% increase in age results in a 15% increase in AUC of FBAL.

Hepatic Insufficiency

Capecitabine has been evaluated in 13 patients with mild to moderate hepatic dysfunction due to liver metastases defined by a composite score including bilirubin, AST/ALT and alkaline phosphatase following a single 1255 mg/m^2 dose of capecitabine. Both AUC_{0-} and C_{max} of capecitabine increased by 60% in patients with hepatic dysfunction compared to patients with normal hepatic function (n=14). The AUC_{0-} and C_{max} of 5-FU was not affected. In patients with mild to moderate hepatic dysfunction due to liver metastases, caution should be exercised when capecitabine is administered. The effect of severe hepatic dysfunction on capecitabine is not known.

Renal Insufficiency

Capecitabine is contraindicated in patients with severe renal impairment (creatinine clearance below 30 mL/min [Cockroft and Gault]). In patients with moderate renal impairment (creatinine clearance 30-50 mL/min [Cockroft and Gault]) at baseline, a dose reduction to 75% of the Capecitabine starting dose is recommended. In patients with mild renal impairment (creatinine clearance 51-80 mL/min) no adjustment in starting dose is recommended.

Cockroft and Gault Equation:

$$\text{Creatinine clearance for males} = \frac{(140 - \text{age [yrs]})\ (\text{body wt [kg]})}{(72)\ (\text{serum creatinine [mg/dL]})}$$

Creatinine clearance for females = 0.85 x male value

Following oral administration of 1250 mg/m^2 capecitabine twice a day to cancer patients with

varying degrees of renal impairment, patients with moderate (creatinine clearance = 30-50 mL/min) and severe (creatinine clearance <30 mL/min) renal impairment showed 85% and 258% higher systemic exposure to FBAL on day 1 compared to normal renal function patients (creatinine clearance >80 mL/min). Systemic exposure to 5'-DFUR was 42% and 71% greater in moderately and severely renal impaired patients, respectively, than in normal patients. Systemic exposure to capecitabine was about 25% greater in both moderately and severely renal impaired patients.

1.5.10 Capecitabine Safety Profile

Hand-and-Foot Syndrome

Hand-and-foot syndrome (palmar-plantar erythrodysesthesia or chemotherapy induced acral erythema) is a cutaneous toxicity (median time to onset of 79 days, range from 11 to 360 days) with a severity range of grades 1 to 3. Grade 1 is characterized by any of the following: numbness, dysesthesia/paresthesia, tingling, painless swelling or erythema of the hands and/or feet and/or discomfort which does not disrupt normal activities. Grade 2 hand-and-foot syndrome is defined as painful erythema and swelling of the hands and/or feet and/or discomfort affecting the patient's activities of daily living. Grade 3 hand-and-foot syndrome is defined as moist desquamation, ulceration, blistering or severe pain of the hands and/or feet and/or severe discomfort that causes the patient to be unable to work or perform activities of daily living. If grade 2 or 3 hand-and-foot syndrome occurs, administration of capecitabine should be interrupted until the event resolves or decreases in intensity to grade 1. Following grade 3 hand-and-foot syndrome, subsequent doses of capecitabine should be decreased.

Cardiotoxicity

The cardiotoxicity observed with capecitabine includes myocardial infarction/ischemia, angina, dysrhythmias, cardiac arrest, cardiac failure, sudden death, electrocardiographic changes, and cardiomyopathy. These adverse events may be more common in patients with a prior history of coronary artery disease.

Hepatic Insufficiency

Patients with mild to moderate hepatic dysfunction due to liver metastases should be carefully monitored when capecitabine is administered. The effect of severe hepatic dysfunction on the disposition of capecitabine is not known.

1.5.11 Adverse Reactions

A total of 596 patients with metastatic colorectal cancer were treated with 1250 mg/m^2 twice a day

of capecitabine administered for 2 weeks followed by a 1-week rest period, and 593 patients were administered 5-FU and leucovorin in the Mayo regimen (20 mg/m^2 leucovorin IV followed by 425 mg/m^2 IV bolus 5-FU, on days 1-5, every 28 days). In the pooled colorectal database the median duration of treatment was 139 days for capecitabine-treated patients and 140 days for 5-FU/LV treated patients. A total of 78 (13%) and 63 (11%) capecitabine and 5-FU/LV-treated patients, respectively, discontinued treatment because of adverse events/intercurrent illness. A total of 82 deaths due to all causes occurred either on study or within 28 days of receiving study drug: 50 (8.4%) patients randomized to capecitabine and 32 (5.4%) randomized to 5-FU/LV.

1.5.12 Combination of Capecitabine and Erlotinib

A phase II study of erlotinib (150 mg daily) with capecitabine (1000 mg/m^2 twice daily) in metastatic colorectal cancer showed grade 3-4 diarrhea in 3 of 10 patients, grade 3-4 renal insufficiency in 1 of 10 patients, and grade 3-4 hyperbilirubinemia in 1 out of 10 patients (Nakhoul, Proc GI Symposium 2006). A phase II study of FOLFOX (5-FU and oxaliplatin), bevacizumab (5 mg/kg) and erlotinib (150 mg daily) showed 31% rate of grade 3-4 diarrhea (Spigel, Proc GI Symposium 2006). Of note, a phase I trial of the EGFR inhibitor gefitinib, capecitabine and radiation therapy showed dose limiting toxicities in 8 out of 16 patients, with the main dose limiting toxicities being diarrhea and arterial thrombosis [23].

1.6 Study Rationale

Definitive chemoradiation with fluoropyrimidine-based chemotherapy is the current standard for unresectable pancreatic adenocarcinoma. Bevacizumab has been studied extensively for anti-angiogenic therapy in pancreatic cancers. Willett *et al.* investigated the addition of bevacizumab to 5-FU for the pre-operative treatment of rectal cancer, and found that bevacizumab decreased tumor perfusion, vascular volume and microvascular density in rectal tumors [11]. At M.D. Anderson, we have successfully completed a phase I trial of preoperative radiotherapy with concurrent bevacizumab and capecitabine for pancreatic cancer [12]. We would like to build upon this experience by adding erlotinib to this regimen.

Erlotinib is an oral reversible inhibitor of EGFR tyrosine kinase. Erlotinib increases survival in patients with locally advanced or metastatic non small cell lung cancer who have failed prior chemotherapy [20]. The addition of erlotinib to gemcitabine has been shown to increase survival and progression free survival in patients with advanced pancreatic cancer [22]. In addition, there is preclinical evidence of synergy between oral tyrosine kinase inhibitors of EGFR and fluoropyrimidines [24]. We believe that simultaneously targeting VEGF and EGFR will lead to

increased response rates in patients with pancreatic cancer. Hence, we are proposing a phase I trial of erlotinib and bevacizumab, in combination with capecitabine-based definitive chemoradiation for pancreatic cancer patients.

2.0 Objectives

2.1 Primary

To evaluate the safety of adding bevacizumab and erlotinib to capecitabine-based chemoradiation in patients with locally advanced pancreatic cancer.

2.2 Secondary

2.2.1 To evaluate whether the addition of bevacizumab and erlotinib to capecitabine-based chemoradiation increases the response rate or the rate of margin-negative resections in patients deemed unresectable at initiation of treatment.

2.2.2 To evaluate the quality of life in patients receiving this therapy.

2.2.3 To evaluate cytokines and VEGF serum levels before, during and after therapy.

3.0 Study Design

3.1 Description of the Study

Phase I study to evaluate the safety of the combination regimen. The doses of capecitabine, bevacizumab and erlotinib will be escalated as shown in Table 1 below. We will use the continual reassessment method to evaluate safety.

Table 1: Dose escalation schema

Dose Level	Bevacizumab Dose (mg/Kg every 2 weeks)	Capecitabine Dose (mg/m^2 bid) XRT days only	Erlotinib Dose (mg QD)	XRT Dose (Gy) GTV only
1	5.0	400	100	50.4
2	5.0	650	100	50.4
3	5.0	650	150	50.4
4	5.0	825	150	50.4
5	10.0	825	150	50.4

3.2 Rationale for Study Design

In the recently completed phase I study at M.D. Anderson, pancreatic cancer patients were treated with preoperative radiotherapy with concurrent bevacizumab (5 mg/kg IV q 2 wks) and capecitabine (825 mg/m2 PO BID). Concurrent bevacizumab did not significantly increase the

acute toxicity of a relatively well-tolerated chemoradiotherapy regimen. Nine (20%) of 46 assessable patients had confirmed partial responses until distant progression for a median of 6.2 months. Four patients underwent pancreaticoduodenectomy without perioperative complication. The median survival was 11.6 months (95% CI, 9.6 to 13.6), from the start of protocol therapy. We propose to investigate the addition of erlotinib to this combination.

Erlotinib, capecitabine and radiotherapy can each cause gastrointestinal toxicity. To reduce the risk of gastrointestinal toxicity, at the first dose level, we will start with a lower dose of capecitabine (400 mg/m2 PO BID) than what was used in the prior study. At the second dose level, the capecitabine dose will be increased, at the third dose level, the dose of erlotinib will be increased and at the fourth dose level, the dose of capecitabine will be increased. Finally, at the fifth dose level, the dose of bevacizumab will be increased.

3.3 Outcome Measures

3.3.1 Primary Outcome Measures

3.3.1.1 The primary endpoint is toxicity. Toxicity will be evaluated using the NCI common toxicity criteria v3.0.

3.3.2 Secondary Outcome Measures

3.3.2.1 Secondary endpoints will include response rate, rate of margin-negative resections in patients deemed unresectable at initiation of treatment, disease-free survival and overall survival.

3.3.3 Safety Outcome Measures

The NCI common toxicity criteria will be used to assess toxicity.

4.0 Safety Plan

4.1 General Plan to Manage Safety

Bevacizumab-Specific

A number of measures will be taken to ensure the safety of patients participating in this trial. These measures will be addressed through exclusion criteria and routine monitoring as follows. Patients enrolled in this study will be evaluated clinically and with standard laboratory tests before and at regular intervals during their participation in this study. Safety evaluations will consist of medical interviews, recording of adverse events, physical examinations, blood pressure, and laboratory measurements. Patients will be evaluated for adverse events (all grades), serious adverse events, and adverse events requiring study drug interruption or discontinuation at each

study visit for the duration of their participation in the study. Patients discontinued from the treatment phase of the study for any reason will be evaluated ~30 days (28–42 days) after the decision to discontinue treatment (see Section 9.1). Specific monitoring procedures are as follows:

- Hypertension will be monitored through routine evaluation of blood pressure prior to each bevacizumab treatment. Optimal control of blood pressure according to standard public health guidelines is recommended for patients on treatment with or without bevacizumab.
- In patients with bleeding, hemostasis evaluation should be performed as clinically indicated.
- Proteinuria will be monitored through urine dipstick at least every 6 weeks.
- If patients on treatment with bevacizumab require elective major surgery, it is recommended that the bevacizumab be held for 4 to 8 weeks prior to the surgical procedure. Patients undergoing a major surgical procedure should not begin/restart bevacizumab until 4 weeks after that procedure (in the case of high-risk procedures such as liver resection, thoracotomy, or neurosurgery, it is recommended that chemotherapy be restarted no earlier than 6 weeks and bevacizumab no earlier than 8 weeks after surgery).
- Patients who have an ongoing bevacizumab-related Grade 4 or serious adverse event at the time of discontinuation from study treatment will continue to be followed (see Section 9.1).
- In patients with suspected RPLS, MRI will be the preferred diagnostic modality, typically demonstrating vasogenic edema predominantly involving the white matter of posterior parietal and occipital lobes, although gray matter and abnormalities in the anterior distributions may also be seen. RPLS is often reversible after correction of the underlying conditions, but in more severe cases, permanent neurological deficit may ensue. Bevacizumab will be held in patients with symptoms/signs suggestive of RPLS, pending work-up and management, including control of blood pressure. Bevacizumab should be discontinued upon diagnosis of RPLS.

Erlotinib-Specific

Common adverse events associated with erlotinib administration include rash and diarrhea. Other common adverse events include nausea/vomiting, stomatitis, headache, and fatigue.

A rash occurred in 75% of erlotinib-treated NSCLC patients enrolled in BR.21. A papular, pustular rash manifesting most often on the face and upper trunk was common across all studies, but rash was rarely the cause of study drug discontinuation. The rash may be associated with erythema, pain, pruritus, dryness, and less commonly, stomatitis, keratitis and nailbed changes. Wearing of contact lenses while receiving erlotinib therapy is not recommended. The incidence of diarrhea in BR.21 was 54% of erlotinib-treated NSCLC patients. The median time to onset of

skin rash was 8 days and median time to occurrence of first diarrheal symptom was 9 days.

There have been infrequent reports of serious ILD (including fatal) in patients receiving erlotinib for treatment of NSCLC or other advanced solid tumors. In Study BR.21, the incidence of ILD (0.8%) was the same in the placebo and erlotinib groups. The overall incidence in erlotinib-treated patients from all studies (including uncontrolled studies and studies with concurrent chemotherapy) is approximately 0.6%. Included in this rate of ILD are reported diagnoses of pneumonitis, interstitial pneumonia, interstitial lung disease, obliterative bronchiolitis, pulmonary fibrosis, acute respiratory distress syndrome, and lung infiltration, irrespective of investigator assessed causality. Most of the cases were associated with confounding or contributing factors such as concomitant/prior chemotherapy, prior radiotherapy, preexisting parenchymal lung disease, metastatic lung disease, or pulmonary infections.

Although quite rare, ILD can be life threatening. Therefore, patients should be monitored closely for symptoms consistent with ILD, such as new onset dyspnea without an obvious cause. In the event that ILD is suspected, erlotinib treatment should be discontinued and the patient should receive appropriate medical management. Although there is no proven therapy, systemic corticosteroids are often provided. erlotinib should not be restarted in those patients suspected of having drug-related ILD. See Section 7.3.4 and Table 5 for management guidelines, including erlotinib dose interruption.

Reversible renal impairment has been reported in association with dehydration associated with nausea, vomiting, and diarrhea. There have been rare reports of renal failure in patients receiving erlotinib in combination with platinum-containing chemotherapy regimens. Febrile neutropenia has been reported in patients receiving concomitant chemotherapy.
Erlotinib is both protein bound (92%–95%) and metabolized by hepatic cytochromes CYP3A4 and CYP3A5 and pulmonary cytochrome CYP1A1. Therefore, a potential for drug–drug interaction exists when erlotinib is co-administered with drugs that are highly protein bound or that are CYP3A4 inhibitors/inducers.

Co-administration of erlotinib with an inhibitor of CYP3A4 metabolism (ketoconazole, 200 mg po BID for 5 days) resulted in increased exposure to erlotinib as measured by an 86% increase in median erlotinib AUC and a 69% increase Cmax, compared with administration of erlotinib alone.
Induction of CYP3A4 metabolism by a known enzyme inducer (rifampin, 600 mg po QD for 7 days) resulted in a 69% decrease in the median Erlotinib AUC, compared with administration of erlotinib alone. However, the effect of rifampin on Cmax was negligible.
International normalized ratio (INR) elevations and/or bleeding events have been reported in

some cancer patients taking warfarin while on Erlotinib. Patients taking warfarin or other warfarin-derivative anticoagulants should be monitored regularly for changes in prothrombin time or INR.

Skin toxicities will be monitored by routine physical examination and managed symptomatically. Because secondary bacterial infections are common and can lead to more serious complications, topical, or systemic antibiotics may be considered. Anecdotally, topical or a short course of systemic corticosteroids can be helpful. See Section 7.3.4 and Table 5 for management guidelines, including erlotinib dose reduction/interruption.

Diarrhea will be monitored and managed symptomatically. Guidelines for management include administration of loperamide and Erlotinib dose reduction/interruption as described in Section 7.3.4 and Table 5.

Patients who have an ongoing erlotinib-related Grade 4 or serious adverse event at the time of discontinuation from study treatment will continue to be followed (see Section 9.1).

Other Study Treatment-Specific
Please see Section 7 for detailed instructions for the management of study drug–related toxicities.

Lymphopenia is an expected result of radiation therapy, has no clinical manifestation, and will not be considered an adverse event.

5.0 Eligibility Criteria

5.1 Inclusion Criteria

5.1.1 ECOG performance status of 0 or 1.

5.1.2 Patients must be $\geq$ 18 years of age. There will be no upper age restriction.

5.1.3 Cytologic or histologic proof of adenocarcinoma of the pancreas. Patients can have tumor originating in any part of the pancreas. Islet cell tumors are not eligible. Only patients with non-metastatic, unresectable disease (AJCC 2002 stage T4 NX M0) are eligible. Patients who cannot undergo resection because of underlying medical problems are also eligible. Patients with regional nodal disease are eligible.

5.1.4 All patients must be staged with a physical exam, CXR, and contrast-enhanced helical thin-cut abdominal CT. Unresectability is defined by CT criteria: a) evidence of tumor extension to the celiac axis or superior mesenteric (SM) artery, or b) evidence on either CT or angiogram of occlusion of the SM vein or SM/ portal vein confluence. If a tumor does not meet this definition and is found to be unresectable at surgical exploration, then that tumor is considered

and is found to be unresectable at surgical exploration, then that tumor is considered unresectable.

5.1.5 Patients may have received prior chemotherapy but not prior radiation therapy to the upper abdomen.

5.1.6 Bone marrow function: absolute neutrophil count (ANC) >1,500/ul. Platelets >100,000/ul.

5.1.7 Hepatic function: Total bilirubin less than 5mg/dL. If the patient required an endobiliary stent, the bilirubin level must have declined on consecutive measurements indicating adequate biliary decompression; alanine aminotransferase (ALT) $\leq$ 5 times the upper limit of normal.

5.1.8 Renal function: BUN $\leq$ 30 mg%, creatinine $\leq$ 1.5 mg% and creatinine clearance $\geq$ 30ml/min (estimated as calculated with Cockcroft-Gault equation). *Note: In patients with moderate renal impairment (estimated creatinine clearance 30-50 mL/min) at baseline, a dose reduction to 75% of the capecitabine starting dose is recommended.*

5.1.9 Patients must have signed informed consent indicating that they are aware of the investigational nature of the study, and are aware that participation is voluntary.

5.2 Exclusion Criteria

5.2.1 Prior abdominal radiotherapy.

5.2.2 Imaging (CT or MRI) or endoscopic evidence of direct duodenal invasion by tumor.

5.2.3 Prior therapy with bevacizumab, cetuximab, or gefitinib. Prior therapy with erlotinib is permitted unless the patient was taken off erlotinib due to treatment failure.

5.2.4 Current, recent (within 4 weeks of the first infusion of this study), or planned participation in any other experimental drug study.

5.2.5 Prior severe infusion reaction (bronchospasm, stridor, urticaria and/or hypotension) to a monoclonal antibody.

5.2.6 Prior unanticipated severe reaction to fluoropyrimidine therapy or known hypersensitivity to 5–fluorouracil.

5.2.7 Proteinuria at baseline or clinically significant impairment of renal function as demonstrated by urine dipstick for proteinuria $\geq$ 2+ (patients discovered to have $\geq$ 2+ proteinuria on dipstick urinalysis at baseline should undergo a 24 hour urine collection and must demonstrate $\leq$ 1g of protein in 24 hours to be eligible).

5.2.8 Prior history of cancer within the last five years except for basal cell carcinoma of the skin or carcinoma in situ of the cervix. Patients with previous malignancies but without evidence of disease for 5 years will be allowed to enter the trial.

5.2.9 Pregnant or lactating women. Women of childbearing potential with either a positive or no pregnancy test at baseline. Women / men of childbearing potential not using a reliable contraceptive method (oral contraceptve, other hormonal contraceptive, intrauterine device, diaphragm or condom). (Postmenopausal women must have been amenorrheic for at least 12 months to be considered of non-childbearing potential). Patients must agree to continue contraception for 30 days from the date of the last study drug administration.

5.2.10 Serious, uncontrolled, concurrent infection(s) requiring IV antibiotics or nonmalignant medical illnesses that are uncontrolled or whose control may be jeopardized by the complications of this therapy.

5.2.11 Uncontrolled hypertension [blood pressure of >140/90 mmHg on medication], New York Heart Association (NYHA) Class II or greater congestive heart failure (see Appendix E), unstable symptomatic arrhythmia requiring medication (subjects with chronic atrial arrhythmia, i.e., atrial fibrillation or paroxysmal supraventricular tachycardia are eligible), significant vascular disease (e.g., aortic aneurysm, aortic dissection) or Class II or greater peripheral vascular disease (see Appendix E), history of stroke or TIA within 6 months prior to study enrollment, history of hypertensive crisis or hypertensive encephalopathy. History of active angina or myocardial infarction within 6 months. History of significant ventricular arrhythmia requiring medication with antiarrhythmics, or a history of a clinically significant conduction system abnormality.

5.2.12 Psychiatric disorders rendering patients incapable of complying with the requirements of the protocol.

5.2.13 History or evidence upon physical examination of CNS disease (e.g., primary brain tumor, seizures not controlled with standard medical therapy, any brain metastases, or history of stroke)

5.2.14 Prior history of pulmonary embolism or deep venous thrombosis.

5.2.15 Major surgical procedure, open biopsy, or significant traumatic injury within 28 days prior to Day 0, or anticipation of need for major surgical procedure during the course of the study, other than that defined by protocol; fine needle aspirations or core biopsies within 7 days prior to Day 0.

5.2.16 Lack of physical integrity of the upper gastrointestinal tract, malabsorption syndrome or inability to swallow.

5.2.17 Known, existing uncontrolled coagulopathy, INR $\geq$ 1.5.

5.2.18 Patients on Coumadin must be changed to Lovenox at least 1 week prior to starting capecitabine. Low dose (1 mg) Coumadin is allowed. Intravenous and low-molecular weight heparin are permitted.

5.2.19 Patients taking Sorivudine or Brivudine must be off of these drugs for 4 weeks prior to starting capecitabine. Patients taking cimetidine must have this drug discontinued. Ranitidine or a drug from another anti-ulcer class can be substituted for cimetidine if necessary. If patient is currently receiving allopurinol, must discuss with PI to see of another agent may substitute for it.

5.2.20 Current serious, nonhealing wound, ulcer, or bone fracture.

5.2.21 History of abdominal fistula, gastrointestinal perforation, or intra-abdominal abscess within 6 months prior to Day 0.

5.2.22 Patients who have had an organ allograft.

5.2.23 Inability to comply with study and/or follow-up procedures.

6.0 Study Schema

6.1 Treatment Plan

Weeks 1 through 6

Pancreatic XRT (50.4 Gy / 28 fractions) (GTV only)

+

Bevacizumab 5 mg/kg or 10 mg/kg IV q2wks x 3 doses*

+

Capecitabine 400 mg/m2, 650 mg/m2 or 825 mg/m2 po bid Mon-Fri x 5.5 weeks*

+

Erlotinib 100 mg or 150 mg po qd x 5.5 weeks*

Weeks 7 through 11-13

No active therapy

Week 11-13

Response assessment with CT/labs

Surgical Evaluation

Week 11-15

Margin-negative resection with maintenance therapy until disease progression at the discretion of the treating medical oncologist

or

Maintenance Bevacizumab and erlotinib until disease progression

(at the same dose-level as that administered during chemoradiation)

*The doses of Bevacizumab, Capecitabine and Erlotinib will be escalated, based on dose level. See Section 3.1, Table 1.

6.2 Patient Monitoring and Supportive Care

Patients will be evaluated weekly by clinicians participating in their care. The principal investigator will be responsible for performing a history, physical exam, and monitoring lab work. At M.D. Anderson, the common acute effects of abdominal chemoradiation are managed with aggressive outpatient supportive care, using outpatient IV hydration, prophylactic antiemetics, a three-step plan to manage diarrhea (Lomotil p.r.n., Lomotil every 3-4 hours, Lomotil alternating with Imodium) and a standardized questionnaire that assesses symptoms (M. D. Anderson Symptom Assessment Inventory).

7.0 Study Treatment

7.1 Bevacizumab dosage and formulation

7.1.1 Bevacizumab Dosage

Bevacizumab will be administered intravenously every 2 weeks +/- 2 days at 5 mg/kg. The first

Bevacizumab will be administered intravenously every 2 weeks +/- 2 days at 5 mg/kg. The first dose will be given on day one of radiotherapy. A total of 3 doses will be given during radiotherapy. In the last cohort of patients, bevacizumab will be administered intravenously every 2 weeks +/- 2 days at 10 mg/kg. Maintenance therapy with bevacizumab administered intravenously every 2 weeks +/- 2 days at 10 mg/kg will be administered with erlotinib thereafter for patients who do not undergo margin-negative curative resections.

7.1.2 Bevacizumab Formulation

Bevacizumab is a clear to slightly opalescent, colorless to pale brown, sterile liquid concentrate for solution for intravenous (IV) infusion. Bevacizumab may be supplied in 5-cc (100-mg), 20-cc (400-mg), and 50-cc (1000-mg) glass vials containing 4 mL, 16 mL, or 40 mL of bevacizumab, respectively (all at 25 mg/mL). Vials contain bevacizumab with phosphate, trehalose, polysorbate 20, and Sterile Water for Injection (SWFI), USP. Vials contain no preservative and are suitable for single use only.

For further details and molecule characterization, see the bevacizumab Investigator Brochure.

7.1.3 Bevacizumab Administration

Bevacizumab will be diluted in at total volume of 100mL of 0.9% Sodium Chloride Injection, USP. Administration will be as a continuous IV infusion. Anaphylaxis precautions should be observed during study drug administration.

The initial dose will be delivered over 90+/-15 minutes. If the first infusion is tolerated without infusion-associated adverse events (fever and/or chills), the second infusion may be delivered over 60+/-10 minutes. If the 60-minute infusion is well tolerated, all subsequent infusions may be delivered over 30+/-10 minutes.

If a subject experiences an infusion–associated adverse event, he or she may be premedicated for the next study drug infusion; however, the infusion time may not be decreased for the subsequent infusion. If the next infusion is well tolerated with premedication, the subsequent infusion time may then be decreased by 30+/-10 minutes as long as the subject continues to be premedicated. If a subject experiences an infusion-associated adverse event with the 60-minute infusion, all subsequent doses should be given over 90+/-15 minutes. Similarly, if a subject experiences an infusion-associated adverse event with the 30-minute infusion, all subsequent doses should be given over 60+/-10 minutes.

7.1.4 Bevacizumab Storage

Upon receipt of the study drug, vials are to be refrigerated at 2C–8C (36F–46F) and should remain refrigerated until just prior to use. DO NOT FREEZE. DO NOT SHAKE. Vials should be protected from light.

Opened vials must be used within 8 hours. VIALS ARE FOR SINGLE USE ONLY. Vials used for 1 subject may not be used for any other subject. Once study drug has been added to a bag of sterile saline, the solution must be administered within 8 hours.

7.1.5 Bevacizumab Dose Modification and Toxicity Management

There are no reductions in the bevacizumab dose. If adverse events occur that require holding bevacizumab, the dose will remain the same once treatment resumes.

Any toxicities associated or possibly associated with bevacizumab treatment should be managed according to standard medical practice. Bevacizumab has a terminal half-life of 2 to 3 weeks; therefore, its discontinuation results in slow elimination over several months. There is no available antidote for bevacizumab.

Subjects should be assessed clinically for toxicity prior to, during, and after each infusion. If unmanageable toxicity occurs because of bevacizumab at any time during the study, treatment with bevacizumab should be discontinued.

Infusion Reaction: Infusion of bevacizumab should be interrupted for subjects who develop dyspnea or clinically significant hypotension. Subjects who experience a NCI CTCAE v. 3.0 Grade 3 or 4 allergic reaction / hypersensitivity, adult respiratory distress syndrome, or bronchospasm (regardless of grade) will be discontinued from bevacizumab treatment.

The infusion should be slowed to 50% or less or interrupted for subjects who experience any infusion-associated symptoms not specified above. When the subject's symptoms have completely resolved, the infusion may be continued at no more than 50% of the rate prior to the reaction and increased in 50% increments every 30 minutes if well tolerated. Infusions may be restarted at the full rate during the next cycle.

Adverse events requiring delays or permanent discontinuation of bevacizumab are listed in Table 2

Regardless of the reason for holding study drug treatment, the maximum allowable length of treatment interruption is 2 months.

Table 2: Bevacizumab Dose Management Due to Adverse Events

Event	Action to be Taken
**Hypertension**	
No dose modifications for grade 1/2 events	
Grade 3	If not controlled to 150/100 mmHg with medication, discontinue bevacizumab.
Grade 4 (including RPLS (confirmed by MRI) or hypertensive encephalopathy)	Discontinue bevacizumab.
**Hemorrhage**	
No dose modifications for grade 1/2 nonpulmonary and non-CNS events	
Grade ≥ 2 pulmonary or CNS hemorrhage	Discontinue bevacizumab.
Grade 3 nonpulmonary and non-CNS hemorrhage	Subjects who are also receiving full-dose anticoagulation will be discontinued from receiving bevacizumab.<br>All other subjects will have study treatment held until all of the following criteria are met:<br>• The bleeding has resolved and hemoglobin is stable.<br>• There is no bleeding diathesis that would increase the risk of therapy.<br>• There is no anatomic or pathologic condition that significantly increases the risk of hemorrhage recurrence.<br>Subjects who experience a repeat Grade 3 hemorrhagic event will be discontinued from receiving bevacizumab.
Grade 4	Discontinue bevacizumab.
**Venous Thrombosis**	
[Note: Subjects with lung cancer placed on anticoagulant therapy for a thrombotic event should be discontinued from receiving bevacizumab]	
No dose modifications for grade 1/2 events	

Grade 3/ Asymptomatic Grade 4	Hold study drug treatment. If the planned duration of full-dose anticoagulation is <2 weeks, study drug should be held until the full-dose anticoagulation period is over. If the planned duration of full-dose anticoagulation is >2 weeks, study drug may be resumed during the period of full-dose anticoagulation if all of the following criteria are met:<br>• The subject must have an in-range INR (usually between 2 and 3) on a stable dose of warfarin (or other anticoagulant) prior to restarting study drug treatment.<br>• The subject must not have had a Grade 3 or 4 hemorrhagic event while on anticoagulation.<br>• The subject must not have had evidence of tumor involving major blood vessels on any prior CT scan.
Symptomatic Grade 4	Discontinue bevacizumab.
**Arterial Thromboembolic event**<br>(Angina, myocardial infarction, transient ischemic attack, cerebrovascular accident, and any other arterial thromboembolic event)	
Any grade	Discontinue bevacizumab.
**Congestive Heart Failure (Left ventricular systolic dysfunction)**<br>No dose modifications for grade 1/2 events	
Grade 3	Hold bevacizumab until resolution to Grade ≤ 1.
Grade 4	Discontinue bevacizumab.
**Proteinuria**<br>No dose modifications for grade 1/2 events	
Grade 3<br>(UPC> 3.5, urine collection > 3.5 g/24 hr, or dipstick 4+)	Hold bevacizumab treatment until ≤ Grade 2, as determined by either UPC ratio ≤ 3.5 or 24 hr collection ≤ 3.5 g
Grade 4 (nephrotic syndrome)	Discontinue bevacizumab

**GI Perforation**	Discontinue bevacizumab.
**Bowel Obstruction**	
Grade 1	Continue patient on study for partial obstruction NOT requiring medical intervention.
Grade 2	Hold bevacizumab for partial obstruction requiring medical intervention. Patient may restart upon complete resolution.
Grade 3/4	Hold bevacizumab for complete obstruction. If surgery is necessary, patient may restart bevacizumab after full recovery from surgery, and at investigator's discretion.
**Wound dehiscence** requiring medical or surgical therapy	Discontinue bevacizumab.
**Other Unspecified Bevacizumab-Related Adverse Events**	
Grade 3	Hold bevacizumab until recovery to ≤ Grade 1
Grade 4	Discontinue bevacizumab.

7.2 CAPECITABINE DOSAGE AND FORMULATION

7.2.1 Capecitabine Dosage

Capecitabine will be given at 400-825 mg/m^2 PO BID only on days of radiation (Monday through Friday), based on the dose level (see Section 3.1, Table 1). Dose adjustment of capecitabine will be made as needed (see 7.2.5).

7.2.2 Capecitabine Formulation, Packaging and Storage

Capecitabine is a white to off-white crystalline powder with an aqueous solubility of 26 mg/mL at 20ºC. Capecitabine is supplied as biconvex, oblong film-coated tablets for oral administration. Each light peach-colored tablet contains 150 mg capecitabine and each peach-colored tablet contains 500 mg capecitabine. The inactive ingredients in capecitabine include: anhydrous lactose, croscarmellose sodium, hydroxypropyl methylcellulose, microcrystalline cellulose, magnesium stearate and purified water. The peach or light peach film coating contains hydroxypropyl methylcellulose, talc, titanium dioxide, and synthetic yellow and red iron oxides.

Capecitabine is commercially available as 150 mg or 500 mg tablets. Capecitabine tablets are packed in polyethylene bottles, containing either 120 x 150 mg tablets or 240 x 500 mg tablets. Capecitabine tablets should be stored at room temperature (15 to 30 C) in the container in which

they are provided.

7.2.3 Capecitabine Administration

Capecitabine should be given approximately 12 hours apart (morning and evening), and taken within 30 minutes after the ingestion of food with approximately 200 mls of water (not fruit juices).

7.2.4 Cautions when used with other medications

Coumarin derivatives

Patients taking coumarin-derivate anticoagulants concomitantly with capecitabine will be monitored weekly for alterations in their coagulation parameters (PT or INR). Altered coagulation parameters and/or bleeding have been reported in patients taking capecitabine concomitantly with coumarin-derivative anticoagulants such as warfarin and phenprocoumon. These events occurred within several days and up to several months after initiating capecitabine therapy and, in a few cases, within one month after stopping capecitabine. These events occurred in patients with and without liver metastases. Therefore a potential interaction between capecitabine and coumarin derivatives has been postulated.

Phenytoin

Increased phenytoin plasma concentrations have been reported during concomitant use of Capecitabine with phenytoin, suggesting a potential interaction. Patients taking phenytoin concomitantly with Capecitabine should be monitored weekly for increased phenytoin plasma concentrations and associated clinical symptoms.

Allopurinol

Oxypurinol, a metabolite of allopurinol, can potentially interfere with 5-FU anabolism via orotate phosphoribosyltransferase. Although this was originally used as a strategy to protect normal tissues from 5-FU-associated toxicity, further laboratory studies suggested possible antagonism of the anticancer activity of 5-FU in some tumor models. If a patient is receiving allopurinol, the need for taking this medicine should be ascertained. If possible, allopurinol should be discontinued prior to starting on this regimen, and another agent substituted for it.

Cimetidine

Because cimetidine can decrease the clearance of 5-FU, patients should not enter on this study until the cimetidine is discontinued. Ranitidine or a drug from another anti-ulcer class can be substituted for cimetidine if necessary.

Sorivudine and Brivudine

A metabolite of the above two investigational antiviral agents, 5-bromovinyluracil, is a potent inhibitor of dihydropyrimidine dehydrogenase, the enzyme that catabolizes 5-FU. Patients should not receive concurrent therapy with either of these antiviral agents while receiving capecitabine. If a patient has received prior sorivudine or brivudine, then at least four weeks must elapse before the patient receives capecitabine therapy.

7.2.5 Dose modification guidelines of Capecitabine

Table 3

Dose Modifications:

For any event which is apparent at baseline, the dose modifications will apply according to the corresponding shift in toxicity grade if the investigator feels it is appropriate (e.g. if a patient has grade 1 asthenia at baseline which increases to grade 2 during treatment, this will be considered as a shift of 1 grade and treated as a grade 1 toxicity for dose modification purposes).

Capecitabine treatment interruptions are regarded as lost treatment days and missed doses should not be replaced; the planned treatment schedule should be maintained.

Once a dose has been reduced it should not be increased at a later time.

Toxicity

NCI Grade (**) (Value)

Neutropenia

Capecitabine will be held for >grade 2 neutropenia. Capecitabine can be restarted at 75% of the dose after recovery o neutropenia to grade 1.

Neutropenic fever

(grade 4 neutropenia and grade 2 fever) Interrupt until resolved, then continue at 75% of starting dose.

Other hematological toxicities

No dose reductions or interruptions will be required for anemia as it can be satisfactorily managed by transfusions.

Diarrhea [all; stools/day > pretreatment]

1 (2-3 stools/day) : Maintain dose

2 (4-6 stools/day)Interrupt until grade 0-1, continue according to number of appearances;
- 1st = 75% of starting dose.
- 2nd = 50% of starting dose.
- 3rd = 50% of starting dose.

3 (7-9 stools/day)Interrupt until grade 0-1, continue according to number of appearances;
- 1st = 75% of starting dose.
- 2nd = 50% of starting dose.
- 3rd = 50% of starting dose.

4 (10 stools/day)Interrupt until grade 0-1, then continue at 50% of starting dose.

Hand-Foot Syndrome (see NCI CTC for grading scale)

1Maintain dose

2Interrupt until grade 0-1, then continue at 75% of starting dose.

3Interrupt until grade 0-1, then continue at 50% of starting dose.

4 (not applicable)

Other non-hematological toxicity

For toxicities which are considered by the Investigator unlikely to develop into serious or life–threatening events and which do not result in a delay or interruption of therapy (e.g. alopecia, altered taste etc.), treatment will be continued at the same dose without reduction or interruption.

1 Maintain dose

2 Interrupt until grade 0-1, continue according to number of appearances of same toxicity;

1st = 75% of starting dose.

2nd = 50% of starting dose.

3rd = 50% of starting dose.

3 Interrupt until grade 0-1, continue according to number of appearances of same toxicity;

1st = 75% of starting dose.

2nd = 50% of starting dose.

3rd = 50% of starting dose.

4 Interrupt until grade 0-1, then continue at 50% of starting dose.

**National Cancer Institute Common Toxicity Criteria Version 3.0, see Appendix B.

7.2.6 Special Instructions Regarding Treatment of Toxicity

7.2.6.1 Grade 2/3 Hand–Foot Skin Reaction

Treat symptomatically (recommended use of emollients). The use of vitamin B6 Pyridoxine has been reported to be of possible benefit and is permitted for symptomatic or secondary prophylactic treatment of hand-foot skin reaction.

7.2.6.2 Fever/Infection with or without neutropenia

Capecitabine should be stopped immediately. Appropriate anti-infective therapy should be initiated. When the ANC has recovered to 1,500/mm^3 and fever of infection has resolved, the patient may restart treatment.

7.2.6.3 Diarrhea

A three-step plan to manage diarrhea will be used. The goal will be to keep the frequency of bowel movements to less than four per day. Patients will initially take Lomotil as needed. When that is no longer sufficient to control the increased frequency of bowel movement, patients take 2 lomotil every 3-4 hours, which is step 2. Subsequently, Imodium is added and alternated with Lomotil, which is step 3; 2 tablets of one or the other is taken every 2-3 hours. Delayed and immediate release narcotics will be used at the discretion of the treating physician. Infectious diarrhea must be considered as an etiology, particularly if diarrhea occurs during the first two weeks of radiation. Outpatient intravenous rehydration will be given in patients who become dehydrated.

7.2.7 Warnings and Precautions

7.2.7.1 Renal Insufficiency

Patients with moderate renal impairment at baseline require dose reduction (See section 1.4.9). Patients with mild and moderate renal impairment at baseline should be carefully monitored for adverse events. Prompt interruption of therapy with subsequent dose adjustments will be made if a patient develops a grade 2 to 4 adverse event. Capecitabine is contraindicated in patients with a creatinine clearance of < 30 ml/min.

7.2.7.2 Pregnancy/Nursing

Capecitabine may cause fetal harm when given to a pregnant woman. If the drug is used during pregnancy, or if the patient becomes pregnant while receiving this drug, the patient should be apprised of the potential hazard to the fetus. Women of childbearing potential should be advised to avoid becoming pregnant while receiving treatment with capecitabine. Because of the potential for serious adverse reactions in nursing infants from capecitabine, it is recommended that nursing be discontinued when receiving capecitabine therapy.

7.2.7.3 Coagulopathy

Patients receiving concomitant capecitabine and oral coumarin-derivative anticoagulant therapy should have their anticoagulant response (INR or prothrombin time) monitored frequently in order to adjust the anticoagulant dose accordingly. A clinically important Capecitabine-Warfarin drug interaction was demonstrated in a clinical pharmacology trial. Altered coagulation parameters and/or bleeding, including death, have been reported in patients taking capecitabine concomitantly with coumarin-derivative anticoagulants such as warfarin and phenprocoumon. Postmarketing reports have shown clinically significant increases in prothrombin time (PT) and INR in patients who were stabilized on anticoagulants at the time capecitabine was introduced. These events occurred within several days and up to several months after initiating capecitabine therapy and, in a few cases, within one month after stopping capecitabine. These events occurred in patients with and without liver metastases. Age greater than 60 and a diagnosis of cancer independently predispose patients to an increased risk of coagulopathy.

7.2.7.4 Cardiotoxicity

The cardiotoxicity observed with capecitabine includes myocardial infarction/ischemia, angina, dysrhythmias, cardiac arrest, cardiac failure, sudden death, electrocardiographic changes, and cardiomyopathy. These adverse events may be more common in patients with a prior history of coronary artery disease. This treatment is foreseen as a self-administered out-patient treatment, and in certain circumstances adverse events that could occur, such as diarrhea, or hand-foot syndrome can rapidly become serious. In the case where a patient experiences any toxicity between scheduled visits, the patient will be instructed to contact the clinic as soon as possible, for further directions, discontinuation of study medication, and/or treatment.

7.3 ERLOTINIB DOSAGE AND FORMULATION

7.3.1 Erlotinib Dosage

Erlotinib will be given at 100 or 150 mg PO daily, based on the dose level (see Section 3.1, Table 1). Erlotinib will be given daily throughout the course of chemoradiotherapy. For patients who are unable to undergo margin-negative resection, maintenance therapy with erlotinib given daily at 150mg/day will be administered with bevacizumab thereafter.

7.3.2 Erlotinib Formulation

The oral tablets are conventional, immediate-release tablets containing erlotinib as the

hydrochloride salt. In addition to the active ingredient, erlotinib tablets contain lactose (hydrous), microcrystalline cellulose, sodium starch glycolate, sodium lauryl sulfate, and magnesium stearate.

Tablets containing 25 mg, 100 mg, and 150 mg of erlotinib are available. Each bottle contains 30 tablets.

7.3.3 Erlotinib Administration and Storage

Erlotinib will be self-administered in an open-label, unblinded manner to all patients enrolled in the study. Tablets should be taken at the same time each day with 200 mL of water at least 1 hour before or 2 hours after a meal. Patients who are unable to swallow tablets may dissolve the tablets in distilled water for administration.

Dose reductions for adverse events will be permitted (see Section 7.3.4). Treatment is continued daily until disease progression or other reason for termination of study therapy (see Sections 9.1 and 9.2).

Erlotinib tablets will be supplied for clinical trials in white, high-density polyethylene (HDPE) bottles with child-resistant closures and should be stored at temperatures between 15°C and 30°C (59°F and 86°F).

7.3.4 Dose Modification Guidelines for Erlotinib

Dose reduction or interruption of erlotinib for toxicity may take place at any time during the study. Toxicity grading is based on NCI-CTCAE, v 3.0. Dose level reductions are presented in the following Table. With a starting dose of 100 mg/day, if patients do not tolerate the second dose reduction, erlotinib is to be discontinued.

Table 4
Erlotinib Dose Level Reductions

Starting Dose	First Reduction	Second Reduction
100 mg/day	50 mg/day	25 mg/day
150 mg/day	100 mg/day	50 mg/day

Dose modification guidelines are summarized in Table 5.

Management of a tolerable Grade 2 or 3 rash should include continuation of erlotinib at the current dose and symptomatic management. If skin rash is intolerable, dose reduction according to Table 5 should be considered. When skin toxicity improves by at least one grade level, the dose may be re-escalated as tolerated. In Phase II trials, this approach enabled dose re-escalation for the majority of patients requiring dose reduction for skin toxicity. Patients experiencing Grade 4 skin toxicity should be discontinued from study treatment.

A three-step plan to manage diarrhea will be used. The goal will be to keep the frequency of bowel movements to less than four per day. Patients will initially take Lomotil as needed. When that is no longer sufficient to control the increased frequency of bowel movement, patients take 2 lomotil every 3-4 hours, which is step 2. Subsequently, Imodium is added and alternated with Lomotil, which is step 3; 2 tablets of one or the other is taken every 2-3 hours. Delayed and immediate release narcotics will be used at the discretion of the treating physician. Infectious diarrhea must be considered as an etiology, particularly if diarrhea occurs during the first two weeks of radiation. Outpatient intravenous rehydration will be given in patients who become dehydrated.

For Grade 1 or 2 diarrhea, early intervention should include continuation of Erlotinib at the current dose. Grade 2 diarrhea that persists over 48–72 hours, despite optimal medical management, should be managed by dose reduction according to Table 5. Patients experiencing Grade 3 diarrhea should interrupt erlotinib until resolution to Grade 1 and re-start at a reduced dose according to Table 5. Patients should be maintained at the reduced dose without attempt at dose re-escalation. Patients experiencing Grade 4 diarrhea should be discontinued from study treatment.

Erlotinib should not be restarted in those suspected of having drug-related ILD.

Table 5
Dosage Modification Criteria and Guidelines for Management of Erlotinib-Related Toxicities

NCI-CTCAE (v 3.0) Grade	Erlotinib Dose Modification	Guideline for Management
**Diarrhea**		
Grade 1	None	Manage as described above
Grade 2	None (Dose reduction of erlotinib is necessary if diarrhea persists over 48–72 hours despite optimal medical management)	Manage as described above
Grade 3	Interrupt then dose reduce erlotinib. Erlotinib should not be re-escalated.	Interrupt erlotinib until resolution to Grade ≤1, and restart at next reduced dose
Grade 4	Discontinue study treatment.	
**Pulmonary Events if possibly ILD**		
All Grades	Temporarily interrupteErlotinib pending the diagnostic evaluation. If the pulmonary adverse event is assessed as related to erlotinib, discontinue the patient from study treatment.	Unexplained dyspnea, either new or progressive, should be aggressively evaluated.
**Rash**		
Tolerable rash	None	Any of the following: minocycline [a], topical tetracycline, topical clindamycin, topical silver sulfadiazine, diphenhydramine, oral prednisone (short course) at discretion of investigator
Intolerable rash	Consider interruption and or dose reduction if unresponsive to symptomatic management. Re-escalation is allowed.	Manage as described above
Grade 4	Discontinue study treatment.	Manage as described above

[a] Recommended dose: 200 mg po bid (loading dose) followed by 100 mg po bid for 7–10 days.

7.3.5 Concomitant and Excluded Therapies

Use of anti-neoplastic or anti-tumor agents not part of the study therapy, including chemotherapy, radiation therapy, immunotherapy, and hormonal anticancer therapy, is not permitted while participating in this study.

Use of concurrent investigational agents is not permitted.

There are potential interactions between erlotinib and CYP3A4 inhibitors and CYP3A4 promoters. Although caution and careful monitoring are recommended when use of these compounds is necessary, use of these compounds does not exclude patients from participating in this trial (see Appendix for a list of CYP3A4 inhibitors).

7.4 RADIATION THERAPY

7.4.1 Radiation Doses and Technique

Radiation therapy will be delivered with a dose of 50.4 Gy in 1.8 Gy fractions to the gross tumor volume (GTV) including any clinically enlarged lymph nodes. If a 3-D conformal plan is utilized, a block margin of 2 cm in all radial directions and 3 cm in the cranial and caudal directions will be used. Total dose will be prescribed to the 95% isodose line using a 2-4 field technique (megavolt photons or proton therapy may be used). If intensity modulated radiation therapy is utilized, the GTV with margin will be treated a multiple beam technique which assures 95% dose coverage. Radiotherapy will be administered concurrently with oral capecitabine, bevacizumab and erlotinib as noted in the dose escalation design above.

7.4.2 Simulation of Radiation Portals

All patients will undergo simulation on a CT simulator and will have CT-based treatment planning.

7.4.3 Radiation Therapy Dose Modification

Patients who experience life threatening hemorrhage or bowel perforation will have radiotherapy discontinued. Radiotherapy will be interrupted for any treatment related > Grade 2 non-hematologic toxicity until the toxicity resolves to Grade 1, then continued. If radiotherapy is interrupted, then capecitabine and erlotinib will also be interrupted.

7.5 SURGERY

7.5.1 Timing of Surgery

Four to six weeks following therapy patients will be presented again at multi-disciplinary conference and re-evaluated for potential resectability. If deemed respectable, surgery must not take place sooner than 6 weeks after the last dose of bevacizumab.

8.0 Clinical and Laboratory Evaluations

8.1 Pre-Treatment Evaluations

8.1.1 Within 3 months of enrollment: biopsy to confirm adenocarcinoma of the pancreas. Where possible, EGFR staining on the biopsy material.

8.1.2 Within 30 days of enrollment: Complete history and physical exam, including blood pressure and other vital signs.

8.1.3 Within 30 days of enrollment: CT or MRI of the abdomen and pelvis, complete history and physical exam, including ECOG PS, blood pressure and other vital signs.

8.1.4 Within 30 days of enrollment: Chest X-ray to rule-out thoracic metastases. If chest X-ray shows indeterminate findings, then chest CT will be required.

8.1.5 Within 10 days of enrollment: laboratory studies to include Ca 19-9, CEA, bilirubin, alkaline phosphatase, ALT, AST, LDH, BUN, creatinine, phosphorus, calcium, glucose, total protein, albumin, and electrolytes [sodium, potassium, carbon dioxide, and chloride], Complete blood count [CBC: hemoglobin, hematocrit, platelets,WBC with differential

total protein, albumin, and electrolytes [sodium, potassium, carbon dioxide, and chloride], Complete blood count [CBC: hemoglobin, hematocrit, platelets,WBC with differential

blood cell counts (neutrophils, bands, lymphocytes, monocytes, eosinophils, basophils)], PT, and urinalysis (including urine protein: creatinine ratio or urine dipstick [and 24 hour collection if indicated]), MDASI - GI module (Appendix D).

8.1.6 Within 24 hours of enrollment: urine pregnancy test for all women of child bearing potential. Postmenopausal women must have been amenorrheic for at least 12 months.

8.1.7 Blood work (optional) - VEGF levels and cytokine levels.

8.2 Evaluations During CHEMORADIATION THERAPY

During therapy, the patients will be assessed as follows: attending physicians and the oncology nurse will evaluate patients every 5 fractions.

8.2.1 Laboratory studies to include bilirubin, alkaline phosphatase, ALT, AST, LDH, BUN, creatinine, phosphorus, calcium, glucose, total protein, albumin, and electrolytes [sodium, potassium, carbon dioxide, and chloride], Complete blood count (CBC: hemoglobin, hematocrit, platelets,WBC with differential blood cell counts (neutrophils, bands, lymphocytes, monocytes, eosinophils, basophils). Must be drawn and evaluated within 3 days of each dose of bevacizumab.

8.2.2 Vital signs (blood pressure, temperature and pulse rate) each week and prior to bevacizumab administration.

8.2.3 Directed history and physical exams, ECOG PS, PT levels for patients on anti-coagulants, MDASI - GI module (Appendix D).

8.2.4 Urinalysis (including urine protein: creatinine ratio or urine dipstick [and 24 hour collection if indicated]) within 3 days of first and third dose of bevacizumab.

8.2.5 Blood work (optional) - VEGF levels and cytokine levels.

8.3 Post-CHEMORADIATION THERAPY Evaluations

8.3.1 Follow-up 4-6 weeks after chemoradiation treatment:

8.3.1.1 History and physical exam, PS

8.3.1.2 Laboratory studies to include Ca 19-9, CEA, bilirubin, alkaline phosphatase, ALT, AST, LDH, BUN, creatinine, phosphorus, calcium, glucose, total protein, albumin, and electrolytes [sodium, potassium, carbon dioxide, and chloride], Complete blood count (CBC: hemoglobin, hematocrit, platelets, WBC with differential blood cell counts (neutrophils, bands, lymphocytes, monocytes, eosinophils, basophils), and PT.

8.3.1.3 Urinalysis (including urine protein: creatinine ratio or urine dipstick [and 24 hour

8.3.1.3 Urinalysis (including urine protein: creatinine ratio or urine dipstick [and 24 hour collection if indicated])

8.3.1.4 Restaging abdominopelvic CT and chest X-ray

8.3.1.5 MDASI - GI module (Appendix D).

8.3.1.6 Blood work (optional) - VEGF levels and cytokine levels.

8.3.2 During maintenance therapy:

8.3.2.1 Monthly (+/- 1 week):

a) History and physical exam, PS performed by a medical oncologist, surgical oncologist, or radiation oncologist.
b) Laboratory studies to include Ca 19-9, CEA, bilirubin, alkaline phosphatase, ALT, AST, LDH, BUN, creatinine, phosphorus, calcium, glucose, total protein, albumin, and electrolytes [sodium, potassium, carbon dioxide, and chloride], Complete blood count (CBC: hemoglobin, hematocrit, platelets, WBC with differential blood cell counts (neutrophils, bands, lymphocytes, monocytes, eosinophils, basophils), and PT.
c) Urinalysis (including urine protein: creatinine ratio or urine dipstick [and 24 hour collection if indicated]) prior to every alternate bevacizumab administration.
d) Vital signs (blood pressure, temperature and pulse rate) prior to each bevacizumab administration.

8.3.2.2 Every 2 months (+/- 1 week):

a) Chest X-ray and abdominopelvic CT scan.

Table 6: Clinical and Laboratory evaluations

Tests and Procedures	Pre-treatment Evaluation	Weekly during Chemoradiation	Restaging Visit (4-6 wks after treatment ends)	Monthly during maintenance therapy
H & P, ECOG PS, BP	X**	X	X	X
CBC	X*	X	X	X
Serum chemistries	X*	X	X	X
Phenytoin#	X*	X	X	
Prothrombin time/ INR##	X*	X	X	
Urinalysis###	X*	X****	X	X
Blood (optional)	X	X@	X	
MDASI GI module	X	X	X	
Chest X-ray	X**		X	X%
Ca 19-9, CEA	X*		X	X
CT or MRI of Abd/Pelvis	X**		X	X%
Urine pregnancy test	X***			

*within 10 days of enrollment, **within 30 days of enrollment, *** within 24 hours of enrollment in women of childbearing potential, ****within 3 days of dose 1 and 3 of bevacizumab

[#] Only for patients on phenytoin
[##] Only for patients on anti-coagulants
[###] Including urine protein: creatinine ratio or urine dipstick [and 24 hour collection if indicated]
[%] Every two months (+/- 1 week)
[@] Every two weeks during bevacizumab infusion

9.0 Criteria for Removal from the Study

9.1 Criteria for Discontinuing Therapy

Treatment will be discontinued if an unexpected, irreversible toxicity develops. Patients who experience any of the following will be taken off study and treatment with bevacizumab and Erlotinib will be discontinued even if the toxicity resolves, and will be counted in the analysis. Any of these events will be considered a dose-limiting toxicity.

1) Any grade 4 hemorrhage, grade 2 pulmonary or CNS hemorrhage
2) Cardiac arrhythmia
3) Grade 4 congestive heart failure
4) Grade 4 hypertension or reversible posterior leukoencephalopathy syndrome (RPLS)
5) Grade 4 nephrotic syndrome
6) Grade 4 diarrhea
7) Grade 4 rash
8) Pulmonary adverse event related to erlotinib
9) Bowel perforation
10) Symptomatic Grade 4 venous thromboembolic event, or any grade arterial thromboembolic event
11) Wound dehiscence requiring medical or surgical intervention
12) Determination by the investigator that it is no longer safe for the subject to continue therapy

Patients with a second episode of the same toxicity related to bevacizumab or Erlotinib after the first has resolved and the drug has been restarted (any grade, other than grade 1-2 rash, grade 1-2 hypertension or grade 1-2 diarrhea) will have drug discontinued permanently. See 12.0 for reporting requirements. Patients who have an ongoing bevacizumab or erlotinib-related Grade 4 or serious adverse event at the time of discontinuation from study treatment will continue to be followed. If the adverse event is felt to be unrelated to capecitabine, capecitabine will be continued.

If the adverse event is felt to be unrelated to radiotherapy, radiotherapy will be continued, with the following exceptions: Patients who experience life threatening hemorrhage or bowel perforation will have radiotherapy discontinued. Radiotherapy will be interrupted for any treatment related > Grade 2 non-hematologic toxicity until the toxicity resolves to Grade 1, then continued. If radiotherapy is interrupted, then capecitabine and erlotinib will also be interrupted.

9.2 Off-study criteria

Statistical analysis will be based on intention-to-treat, and all patients who begin protocol based therapy

Statistical analysis will be based on intention-to-treat, and all patients who begin protocol based therapy will be counted in the analysis. Patients who have documented clinical progression of disease that is discovered during therapy will be taken off study. Patients will be taken off study if they fail to complete protocol therapy for reasons other than treatment related toxicity or tumor progression.

10.0 Study Discontinuation

The trial will be terminated early if there is more than a 95% chance that the probability of Grade 3 or higher toxicity at the lowest dose level is greater than 0.25, as discussed in the following section.

11.0 Statistical Methods

This is a phase I trial of radiotherapy administered concurrently with 5 dose combinations of capecitabine (400 – 825 mg/m² p.o. bid on days of radiation treatment), bevacizumab (5 mg/kg or 10mg/kg IV q 2 weeks), and Erlotinib (100 mg p.o. qd or 150 mg po qd) in patients with locally advanced pancreatic cancer. The 5 dose combinations are described in section 3.1 of the protocol. After a re-staging 4-6 following chemoradiation, patients who are unable to undergo margin-negative resections will receive maintenance chemotherapy with bevacizumab and erlotinib until progression.

The trial will select from among the 5 dose combinations using the continual reassessment method (CRM) described below [25] to determine the maximum tolerated dose (MTD).

<u>Statistical Considerations</u>

We will enroll a maximum of 30 patients in cohorts of size 2 at a rate of 2-3 patients per month. We will start at the lowest dose, and we will not skip a dose when searching for the MTD among 5 combination doses of bevacizumab, capecitabine, erlotinib, and xrt, as defined elsewhere. *A priori* we assume the toxicity probabilities for the 5 combination doses are $(p_1, p_2, p_3, p_4, p_5) = (0.03, 0.05, 0.15, 0.25, 0.35)$. We assume the exponential model Prob(toxicity at dose level j) = $p_j^{\exp(\alpha)}$, where the parameter α has a normal distribution with mean 0 and variance of 2. The target toxicity probability is 0.25, and we are primarily concerned with gastrointestinal toxicity (diarrhea, nausea, GI bleed, GI ulceration) evaluated upon completion of treatment.

As an added measure of safety the trial will be stopped early if the lowest dose level is unacceptably toxic. That is, if there is more than a 95% chance that the probability of toxicity at the lowest dose level is greater than 0.25, then we will stop the study.

The operating characteristics of the CRM design are illustrated in Table 1. These operating

The operating characteristics of the CRM design are illustrated in Table 1. These operating characteristics are based on 1000 simulations of the trial.[26]

Table 1. Operating Characteristics of CRM Design

	Dose of Combination Therapy					
	1	2	3	4	5	No Dose Selected
P(toxicity)	0.03	0.05	0.15	0.25	0.35	---
P(MTD)	0.00	0.05	0.33	0.42	0.20	0.00
Avg # pts	2.2	4.6	9.0	8.9	5.2	
	Expected Number of Patients with Toxicity is 5.6					
P(toxicity)	0.05	0.15	0.25	0.35	0.45	---
P(MTD)	0.01	0.33	0.47	0.18	0.01	0.00
Avg # pts	2.7	10.6	10.7	4.6	1.4	
	Expected Number of Patients with Toxicity is 6.7					
P(toxicity)	0.00	0.10	0.20	0.30	0.40	---
P(MTD)	0.00	0.12	0.43	0.37	0.08	0.00
Avg # pts	2.1	6.3	11.0	7.6	3.1	
	Expected Number of Patients with Toxicity is 6.3					
P(toxicity)	0.00	0.00	0.05	0.10	0.20	---
P(MTD)	0.00	0.00	0.01	0.16	0.83	0.00
Avg # pts	2.0	2.0	3.1	6.4	16.5	
	Expected Number of Patients with Toxicity is 4.1					
P(toxicity)	0.20	0.25	0.30	0.35	0.40	---
P(MTD)	0.23	0.43	0.20	0.10	0.02	0.02
Avg # pts	9.3	11.8	5.1	2.4	1.0	
	Expected Number of Patients with Toxicity is 7.5					

The secondary endpoint is to evaluate whether the addition of bevacizumab and erlotinib to capecitabine-based chemoradiation increases the response rate or the rate of margin-negative resections in patients deemed unresectable at initiation of treatment.

The following will be considered in the evaluation of tumors:

Lesions that are only reported as one dimension should be re-read and have two dimensional measurements provided. Lesions reported with three dimensions will have the two largest measurements reported and will be followed to determine response as noted below.

- Complete response (CR) — complete disappearance of clinical evidence of a tumor. The patient must be free of all symptoms of cancer. Radiographically equivocal lesions must remain stable or regress.
- Partial response (PR) — 50% or greater decrease in the sum of the products of the longest perpendicular diameters of all measured lesions compared to baseline. No simultaneous increase in the size of any lesions 25% or the appearance of new lesions may occur. Radiographically equivocal lesions must remain stable or regress.
- Stable disease (SD) — no significant change in disease status. Lesions may show a <50% decrease in the sum of products of the longest perpendicular diameters of measured lesions or an increase of <25%. No new lesions may appear.
- Progressive disease (PD) — a 25% increase in the area of malignant lesions >2 cm^2 or in the sum of the products of the longest perpendicular diameters of individual lesions in a given organ site. If only one lesion is available for measurement, a 50% increase in the size if the area of the lesion was 2 cm^2. The appearance of new lesions will also constitute progressive disease. Comparisons of tumor size will be made with the previous smallest measurement in patients who have attained a partial response or with baseline measurements in patients with stable disease. Tumor progression will also be defined as significant clinical deterioration that cannot be attributed to treatment or other medical conditions.

12.0 Safety Reporting of Adverse Events

12.1 Adverse Event Reporting and Definitions

In the event of an adverse event the first concern will be for the safety of the subject.

Investigators are required to report to Genentech Drug Safety ANY serious treatment emergent adverse event (STEAE) as soon as possible.

A STEAE is any sign, symptom or medical condition that emerges during Bevacizumab treatment or during a post-treatment follow-up period that (1) was not present at the start of Bevacizumab treatment and it is not a chronic condition that is part of the patient's medical history, OR (2) was present at the start of Bevacizumab treatment or as part of the patient's medical history but worsened in severity and/or

and it is not a chronic condition that is part of the patient's medical history, OR (2) was present at the start of Bevacizumab treatment or as part of the patient's medical history but worsened in severity and/or frequency during therapy, AND that meets any of the following regulatory serious criteria:

- Results in death
- Is life-threatening
- Requires or prolongs inpatient hospitalization
- Is disabling
- Is a congenital anomaly/birth defect
- Is medically significant or requires medical or surgical intervention to prevent one of the outcomes listed above.

12.2 Reporting of Serious Treatment Emergent Adverse Events

All STEAEs should be recorded on a MedWatch 3500a Form and faxed to:

Genentech Drug Safety
Fax: (650) 225-4682 or (650) 225-4683

(Please use the safety reporting fax cover sheet attached to this document for your fax transmission)

AND:

Study Coordination Center/Principal Investigator
Contact Information and fax #

AND:

IRB Contact information and fax #

MedWatch 3500a Reporting Guidelines:

In addition to completing appropriate patient demographic and suspect medication information, the report should include the following information within the Event Description (section 5) of the MedWatch 3500a form:

- Treatment regimen (dosing frequency, combination therapy)
- Protocol description (and number, if assigned)
- Description of event, severity, treatment, and outcome, if known
- Supportive laboratory results and diagnostics
- Investigator's assessment of the relationship of the adverse event to each investigational product and

- Investigator's assessment of the relationship of the adverse event to each investigational product and suspect medication

Follow-up information:
Additional information may be added to a previously submitted report by any of the following methods:

- Adding to the original MedWatch 3500a report and submitting it as follow-up
- Adding supplemental summary information and submitting it as follow-up with the original MedWatch 3500a form
- Summarizing new information and faxing it with a cover letter including subject identifiers (i.e. D.O.B. initial, subject number), protocol description and number, if assigned, suspect drug, brief adverse event description, and notation that additional or follow-up information is being submitted (The subject identifiers are important so that the new information is added to the correct initial report)

Occasionally Genentech may contact the reporter for additional information, clarification, or current status of the subject for whom and adverse event was reported.

Assessing Causality:

Investigators are required to assess whether there is a reasonable possibility that bevacizumab caused or contributed to an adverse event. The following general guidance may be used.

Yes: if the temporal relationship of the clinical event to bevacizumab administration makes a causal relationship possible, and other drugs, therapeutic interventions or underlying conditions do not provide a sufficient explanation for the observed event.

No: if the temporal relationship of the clinical event to bevacizumab administration makes a causal relationship unlikely, or other drugs, therapeutic interventions or underlying conditions provide a sufficient explanation for the observed event.

12.3 Safety Reporting Requirements for IND Exempt Studies

For **Investigator Sponsored IND Exempt Studies**, there are some reporting requirements for the FDA in accordance with the guidance set forth in 21 CFR 314.80.

Postmarketing 15-Day "Alert Report":
The Sponsor-Investigator is required to notify the FDA of any fatal or life-threatening adverse event that is **unexpected and assessed by the investigator to be possibly related to the use of Bevacizumab**. An unexpected adverse event is one that is not already described in the Investigator Brochure. Such

The Sponsor-Investigator is required to notify the FDA of any fatal or life-threatening adverse event that is **unexpected and assessed by the investigator to be possibly related to the use of Bevacizumab**. An unexpected adverse event is one that is not already described in the Investigator Brochure. Such reports are to be submitted to the FDA (2 copies) at the following address: Central Document Room, 12229 Wilkins Avenue, Rockville, MD 20852.

All Postmarketing 15-Day "Alert Reports" submitted to the FDA by the Sponsor-Investigator must also be faxed to: Genentech Drug Safety

Fax: (650) 225-4682 or (650) 225-4683 (Please use the safety reporting fax cover sheet attached to this document for your fax transmission)

For questions related to safety reporting, contact:

Genentech Drug Safety
Tel: 1-888-835-2555
or
Fax: (650) 225-4682 or (650) 225-4683
(Please use the safety reporting fax cover sheet attached to this document for your fax transmission)

13.0 References

1. Sener SF, Fremgen A, Menck HR, Winchester DP. Pancreatic cancer: a report of treatment and survival trends for 100,313 patients diagnosed from 1985-1995, using the National Cancer Database. *J Am Coll Surg.* 1999;189:1-7.
2. A multi-institutional comparative trial of radiation therapy alone and in combination with 5-fluorouracil for locally unresectable pancreatic carcinoma. The Gastrointestinal Tumor Study Group. *Ann Surg.* 1979;189:205-208.
3. Treatment of locally unresectable carcinoma of the pancreas: comparison of combined-modality therapy (chemotherapy plus radiotherapy) to chemotherapy alone. Gastrointestinal Tumor Study Group. *J Natl Cancer Inst.* 1988;80:751-755.
4. Rich T, Harris J, Abrams R, et al. Phase II study of external irradiation and weekly paclitaxel for nonmetastatic, unresectable pancreatic cancer: RTOG-98-12. *Am J Clin Oncol.* 2004;27:51-56.
5. Burris HA, 3rd, Moore MJ, Andersen J, et al. Improvements in survival and clinical benefit with gemcitabine as first-line therapy for patients with advanced pancreas cancer: a randomized trial. *J Clin Oncol.* 1997;15:2403-2413.

6. Crane CH, Abbruzzese JL, Evans DB, et al. Is the therapeutic index better with gemcitabine-based chemoradiation than with 5-fluorouracil-based chemoradiation in locally advanced pancreatic cancer? *Int J Radiat Oncol Biol Phys.* 2002;52:1293-1302.
7. Hoff PM, Ansari R, Batist G, et al. Comparison of oral capecitabine versus intravenous fluorouracil plus leucovorin as first-line treatment in 605 patients with metastatic colorectal cancer: results of a randomized phase III study. *J Clin Oncol.* 2001;19:2282-2292.
8. Twelves C, Wong A, Nowacki MP, et al. Capecitabine as adjuvant treatment for stage III colon cancer. *N Engl J Med.* 2005;352:2696-2704.
9. Hurwitz H, Fehrenbacher L, Novotny W, et al. Bevacizumab plus irinotecan, fluorouracil, and leucovorin for metastatic colorectal cancer. *N Engl J Med.* 2004;350:2335-2342.
10. Kabbinavar FF, Hambleton J, Mass RD, Hurwitz HI, Bergsland E, Sarkar S. Combined analysis of efficacy: the addition of bevacizumab to fluorouracil/leucovorin improves survival for patients with metastatic colorectal cancer. *J Clin Oncol.* 2005;23:3706-3712.
11. Willett CG, Boucher Y, di Tomaso E, et al. Direct evidence that the VEGF-specific antibody bevacizumab has antivascular effects in human rectal cancer. *Nat Med.* 2004;10:145-147.
12. Crane CH, Ellis LM, Abbruzzese JL, et al. Phase I trial evaluating the safety of bevacizumab with concurrent radiotherapy and capecitabine in locally advanced pancreatic cancer. *J Clin Oncol.* 2006;24:1145-1151.
13. Ozcan C, Wong SJ, Hari P. Reversible posterior leukoencephalopathy syndrome and bevacizumab. *N Engl J Med.* 2006;354:980-982; discussion 980-982.
14. Glusker P, Recht L, Lane B. Reversible posterior leukoencephalopathy syndrome and bevacizumab. *N Engl J Med.* 2006;354:980-982; discussion 980-982.
15. Skillings JR, Johnson DH, Miller K, et al. Arterial thromboembolic events (ATEs) in a pooled analysis of 5 randomized, controlled trials (RCTs) of bevacizumab (BV) with chemotherapy. *J Clin Oncol.* 2005;16S:3019 (abstr).
16. Hambleton J, Skillings J, Kabbinavar F, et al. Safety of low-dose aspirin (ASA) in a pooled analysis of 3 randomized, controlled trials (RCTs) of bevacizumab (BV) with chemotherapy (CT) in patients (pts) with metastatic colorectal cancer (mCRC). *J Clin Oncol.* 2005;23:3554.

17. Scappaticci FA, Fehrenbacher L, Cartwright T, et al. Surgical wound healing complications in metastatic colorectal cancer patients treated with bevacizumab. *Journal of Surgical Oncology.* 2005;91:173-180.

18. Bevacizumab Investigator Brochure 2005.

19. Karp JE, Gojo I, Pili R, et al. Targeting vascular endothelial growth factor for relapsed and refractory adult acute myelogenous leukemias: therapy with sequential 1-beta-d-arabinofuranosylcytosine, mitoxantrone, and bevacizumab. *Clinical Cancer Research.* 2004;10:3577-3585.

20. Shepherd FA, Rodrigues Pereira J, Ciuleanu T, et al. Erlotinib in previously treated non-small-cell lung cancer. *N Engl J Med.* 2005;353:123-132.

21. Herbst RS, Prager D, Hermann R, et al. TRIBUTE: a phase III trial of erlotinib hydrochloride (OSI-774) combined with carboplatin and paclitaxel chemotherapy in advanced non-small-cell lung cancer. *J Clin Oncol.* 2005;23:5892-5899.

22. Moore MJ. Brief communication: a new combination in the treatment of advanced pancreatic cancer. *Semin Oncol.* 2005;32:5-6.

23. Czito BG, Willett CG, Bendell JC, et al. Increased toxicity with gefitinib, capecitabine, and radiation therapy in pancreatic and rectal cancer: phase I trial results. *J Clin Oncol.* 2006;24:656-662.

24. Magne N, Fischel JL, Dubreuil A, et al. ZD1839 (Iressa) modifies the activity of key enzymes linked to fluoropyrimidine activity: rational basis for a new combination therapy with capecitabine. *Clin Cancer Res.* 2003;9:4735-4742.

25. O'Quigley J, Shen LZ. Continual reassessment method: a likelihood approach. *Biometrics.* 1996;52:673-684.

26. O'Quigley J, Pepe M, Fisher L. (1990) Continual reassessment method: A practical design for phase I clinical trial in cancer. Biometrics 46;33-48.
